# Locomotion of an untethered, worm-inspired soft robot driven by a shape-memory alloy skeleton

**DOI:** 10.1038/s41598-022-16087-5

**Published:** 2022-07-20

**Authors:** Lin Xu, Robert J. Wagner, Siyuan Liu, Qingrui He, Tao Li, Wenlong Pan, Yu Feng, Huanhuan Feng, Qingguang Meng, Xiang Zou, Yu Fu, Xingling Shi, Dongliang Zhao, Jianning Ding, Franck J. Vernerey

**Affiliations:** 1 Institute of Intelligent Flexible Mechatronics, Jiangsu University, Zhenjiang, 212013 People’s Republic of China; 2 State Key Laboratory of Solid Lubrication, Lanzhou Institute of Chemical Physics, Chinese Academy of Sciences, Lanzhou, 730000 People’s Republic of China; 3 Department of Mechanical Engineering & Material Science and Engineering Program, University of Colorado at Boulder, Boulder, 80309-0428 USA; 4 School of Materials Science and Engineering, Jiangsu University of Science and Technology, Zhenjiang, 212003 People’s Republic of China; 5 School of Energy and Environment, Southeast University, Nanjing, 210096 People’s Republic of China

**Keywords:** Soft materials, Actuators, Bioinspired materials, Mechanical engineering

## Abstract

Soft, worm-like robots show promise in complex and constrained environments due to their robust, yet simple movement patterns. Although many such robots have been developed, they either rely on tethered power supplies and complex designs or cannot move external loads. To address these issues, we here introduce a novel, maggot-inspired, magnetically driven “mag-bot” that utilizes shape memory alloy-induced, thermoresponsive actuation and surface pattern-induced anisotropic friction to achieve locomotion inspired by fly larvae. This simple, untethered design can carry cargo that weighs up to three times its own weight with only a 17% reduction in speed over unloaded conditions thereby demonstrating, for the first time, how soft, untethered robots may be used to carry loads in controlled environments. Given their small scale and low cost, we expect that these mag-bots may be used in remote, confined spaces for small objects handling or as components in more complex designs.

## Introduction

The recent emergence of robots comprised of flexible materials rather than rigid structures has introduced broad prospects in the field of robotics engineering^1^. Like soft creatures, soft robots are composed of highly deformable materials^1–3^, thereby introducing several advantages over their rigid counterparts. For example, soft robots may deform to the contours of confined spaces and can exhibit continuous, multimodal deformations due to their flexibility, making them highly sought after for traversal of complex mechanical environments^4–9^. Additionally, soft materials are generally safer for human–machine interactions^10^ and exhibit higher biocompatibility^11^, rendering them suitable for both in vivo and in vitro applications^12^. Despite these advantages, there remain shortcomings and challenges pertaining to soft robots’ miniaturization, load carrying capacity, and autonomy. However, nature is rife with living designs that have achieved all three features.

Soft invertebrates such as caterpillars^13,14^, earth worms^15,16^, and larvae of insects in the order *Diptera* (i.e., flies)^17^ have evolved to use peristaltic or wave-like actuation in conjunction with some means of asymmetrical anchoring to move reliably within their environments. These principals of soft actuation and asymmetrical friction have recently inspired many ideas for the design and research of multi-functional and highly adaptable soft, biomimetic robots^18–27^. However, arguably the simplest (and therefore the most size- and cost-scalable) designs utilize alternating modes of bending and extension as their only mechanisms of actuation^18–20^, thus negating the need for complex internal mechanisms, control systems, or localized actuators as in the case of bots that replicate non-harmonic, wave-like peristaltic actuation. Instead, these simple bots may be comprised entirely of one or a few stimuli-sensitive materials^3^. Consequently, such designs also mitigate the need for hydro-pneumatic umbilicals^21–28^ or electrical tethers^29–32^ for their power supply. Furthermore, while more complex systems attempt to induce unidirectional anchoring through the use of legs and adhesive sites (mimicking the case of caterpillars^13^) or localized lateral expansion and contraction (to replicate worms or fly larvae^16,17^), simpler designs aspire to leverage anisotropic surfaces or bot-skin patterns that cause asymmetric friction or ratchet-like motion^18,19,33–35^. These take inspiration from natural systems such as hook-like formations occurring on the skin of fly larvae^17^.

We here introduce a simple and scalable maggot-inspired and magnetically driven robot or “mag-bot”^19^ that utilizes unimodal bending and extension as its only means of actuation. It achieves unidirectional motion via specifically designed interfacing geometries at its head and tail ends. The simplicity of this design derives from its use of a single strip of nickel titanium (NiTi) shape-memory alloy (SMA)^33,36–43^ that extends down the length of the mag-bot and comprises its internal skeleton. The SMA skeleton, which exists in a bent conformation at room temperature, extends upon heating and then bends back to its original shape upon cooling. Temperature changes are induced through alternating magnetic fields that heat the SMA as needed, and which are imparted by alternating current passed through an induction coil. While an external power supply is indispensable in this design, this magnetic induction eliminates the need for any tethers, thereby freeing the mag-bot from physical restraints. In the remainder of this work, we briefly overview the design of the mag-bot, characterize its magnetothermal actuation, and introduce the simple geometric feature that impose asymmetric fiction. We then characterize the anisotropic friction, travel speed, and movement efficiency under different external loads for mag-bots with three different skin pattern angles, highlighting the existence of a biphasic relationship between mag-bot performance and pattern design.

## Results and discussion

### Design of the mag-bot

**Figure 1:**
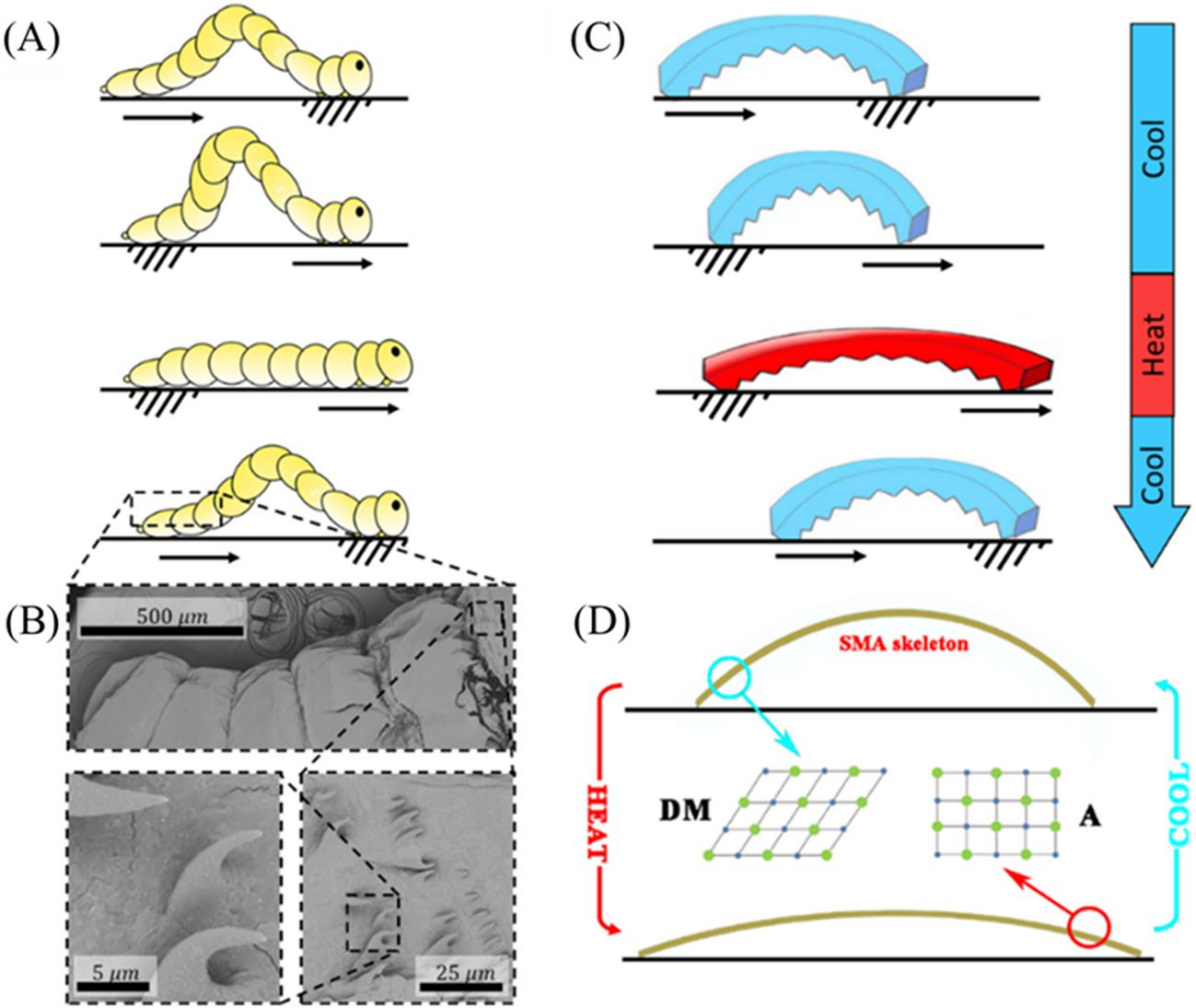
Schematic diagram of mag-bot. (**A**) A schematic of fruit fly larva movement displays the phases of peristaltic motion in which a larva anchors its head, pulls its tail towards its head, anchors hind portions of its body, and then pushes its head forwards. In many organisms^13,17^, such waves propagate non-harmonically along the creatures’ lengths, however—for simplicity—this is not illustrated here. (**B**) Scanning electron microscopic images of the surface microstructure of fruit fly larvae display hook-like features that give rise to asymmetric friction. (**C**) A schematic of ideal, bio-inspired mag-bot motion is displayed under a cycle of heating and cooling. (**D**) A schematic of the SMA skeleton inside of a mag-bot illustrates the microstructural phase evolution within the alloy that drives mag-bot bending and extension as the microstructure alternates between detwinned martensite (DM) and austenite (A) phases. Movement in (**A,C**) is denoted by arrows while anchoring is denoted by hatching.

The movement of a fly larva is driven by cyclical phases in which the larva anchors its head, pulls the directly trailing portions of its body forward, anchors its rear, and then pushes its head forward (Fig. 1A). Anchoring is facilitated by microscopic, spine-like hooks along the length of the larva’s body (see Fig. 1B for SEM images). Directional motion then ensues due to the anisotropic friction between the larvae and the substrate during anchoring. Based on these observations, we fabricated the bioinspired mag-bots according to the procedure outlined in Fig. S1 (see the “Materials and methods” section for details). While true biological peristaltic motion generally involves multi-modal and continuous, wave-like propagation along the length of a larva^17^, our simplified mag-bots have only two contact regimes—a head and tail—at any given moment. This permits the use of a simpler form of harmonic, unimodal bending and extension.

Actuation is achieved via a small SMA skeletal strip embedded in the mag-bot. At low temperature, the SMA skeleton assumes a bent state (Fig. 1C) and is comprised of a detwinned martensite (DM) micro-structure (Fig. 1D). When the temperature is raised, the SMA structure changes from a DM phase to austenite (A) phase (Fig. 1D), which induces mechanical deformation and an overall straightening of the skeleton. This process is reversible, such that a periodic deformation of the skeleton can be achieved by remotely applying an alternating magnetic field, which heats the SMA skeleton. Asymmetric anchoring is achieved through a wedge-shaped, anisotropic interface geometry inspired by the hook-like formations found on fly larvae (Fig. 1B).

### Magnetothermal deformation of the SMA skeleton

**Figure 2:**
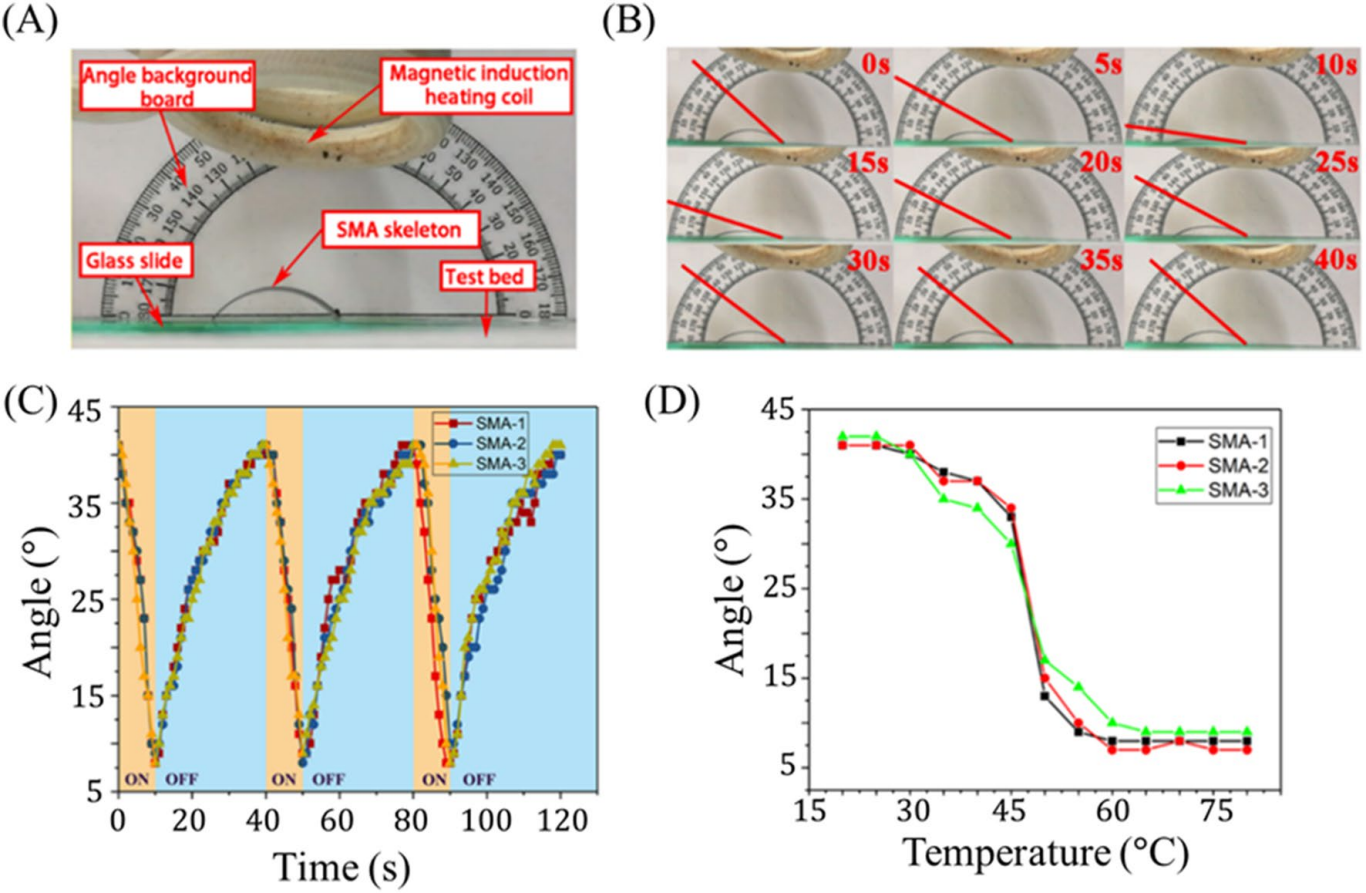
Magnetothermal deformation response of SMA actuator. (**A**) The experimental setup used to measure magnetothermal response of the SMA strip is shown. (**B**) One cycle of periodic deformation is displayed, with the tangent angle used to characterize deformation denoted by a red line. (**C**) Deformation angle is plotted with respect to time for three SMA skeletons undergoing three periods of reversible loading (each). (**D**) Deformation angle is plotted with respect to temperature of the SMA skeleton.

The extent of magnetothermal deformation of the SMA skeleton is characterized in Fig. 2. Figure 2A displays the experimental setup used to quantify deformation. The sample resides on a glass plate, 40 mm above which the induction coil is positioned. To measure the deformation angle of the sample, a protractor is placed in the background so that its center coincides with the right end of the sample, which is fixed in place. Deformation is quantified by the tangent angle to the SMA strip at the position of this fixed right side (Fig. 2B). A larger angle indicates a higher degree of bending (i.e., that the sample is closer to its reference state), while a lower angle indicates a higher degree of extension (i.e., that the sample is further from its reference state).

Figure 2B visually illustrates the measured bending angle at different times as the SMA sample undergoes heating due to magnetic induction. Notably there exists a lower temperature threshold (~ 25 °C) below which additional temperature decrease does not induce further actuation. Likewise, above approximately 60 °C, additional temperature change induces no further extension. The corresponding response angles with respect to time and SMA temperature are displayed in Fig. 2C,D, respectively. For each period of thermal loading, the magnetic field is introduced during the time interval of 0–10 s and then removed for the interval 10–40 s. Correspondingly, the bending angle of the sample, which starts at 41°, decreases to 8° (after 10 s of heating) and then recovers to 37° (after the 30 s of cooling). The bending angle completely and repeatedly recovers after 40 s (Fig. 2B,C) and remains between 9$^\circ$ and 41$^\circ$ throughout the entire cycle, exemplifying the stability and reversibility of this process across samples and load cycles.

We note that during the initial stage (0–5 s) of magnetic heating, the sample only exhibits limited deformation (between 41° and 30°) while during the later stage (5–10 s), larger deformations are observed (from 30° to 9°). Similar trends are observed during recovery where most of the deformation is seen in the initial stage (9° to 37° between 10 and 30 s). In other words, extensile actuation is slow in the early stages and rapid in the later stages while bending recovery is rapid in the early stages and slow in the later stages. This effect is likely influenced by the rate of heating and cooling, and so to better understand actuation control with respect to temperature, we examine Fig. 2D. This plot reveals a sharp change from highly bent (at low temperatures) to weakly bent (at high temperatures) at a temperature around 45–50 °C, indicating an estimated phase transition temperature range for this alloy (0.8 wt% Si, 43.8 wt% Ti, 55.4 wt% Ni—see Fig. S2 for compositional characterization). The detwinned martensite to austenite transition temperature of NiTi SMA is highly sensitive to Ni content in the alloy, reportedly spanning from − 50 to 110 °C for the respective compositional range of 49 to 57 wt% Ni^44^. Therefore, the specific alloy used represents an important design parameter and may be altered to shift the transition temperature for specific applications.

Another important set of design considerations is the geometric traits of the SMA. While only the SMA parameters explored in Fig. 2 were incorporated into full mag-bot designs for this study, we found that actuation properties exhibit good repeatability regardless of the SMA’s initial bend angles, widths, or lengths (Fig. S3). Doubling the initial bend angle slightly reduces the range of tangent angles for the SMA throughout actuation, although whether this amounts to a significant change in horizontal actuation strain also depends largely on the length of the SMA. Increasing the SMA length increases the range of tangent angles, culminating in an increase in horizontal actuation strain. While increasing the SMA width has no conformational effect on the SMA during actuation, it will inevitably increase the actuation load and thus may still impact mag-bot performance. Likewise, increasing the initial bend angle and length both influence the amount of torque experienced by the mag-bot due to horizontal extension, thus also influencing the necessary mag-bot actuation force. Therefore, these parameters introduce complex trade-offs between horizontal actuation strain and actuation force that must be considered when designing for specific applications.

### Magnetothermal deformation of the mag-bot

**Figure 3:**
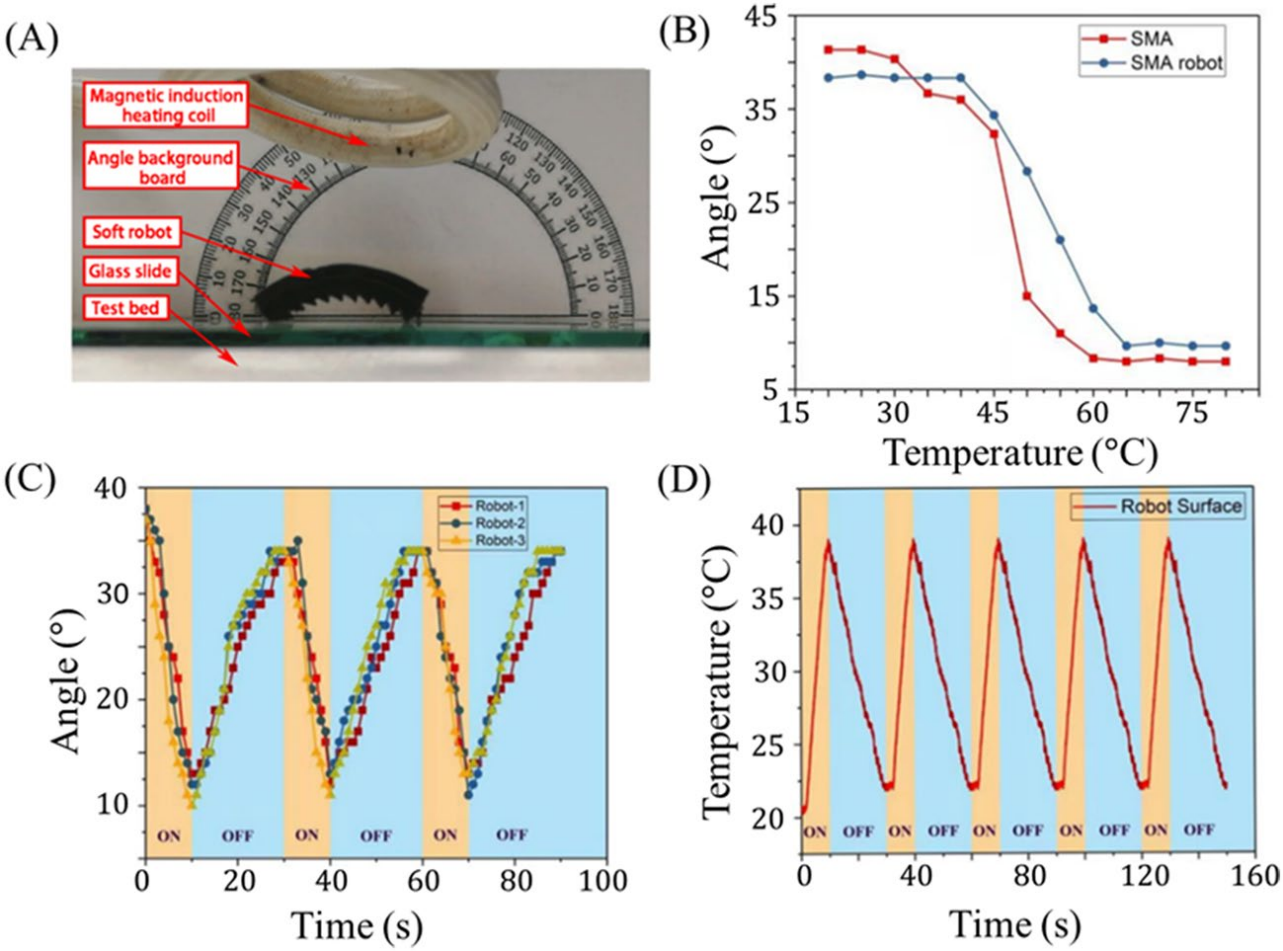
Magnetothermal response behavior of SMA mag-bot. (**A**) The experimental setup used to measure magnetothermal response of the mag-bot is shown. (**B**) The average bending angle response of three mag-bot prototypes (blue circles) and three SMA skeletons (red squares) is plotted with respect to temperature. (**C**) Deformation angle is plotted with respect to time for three mag-bots undergoing three periods of reversible loading (each). (**D**) Average surface temperature of three mag-bots is plotted with respect to time for five periods of thermal loading.

The magnetothermal response of the mag-bot is characterized in Fig. 3. The experimental setup (Fig. 3A) is identical to that used to characterize the deformation of the SMA skeleton (Fig. 2A). Like the SMA sample, the mag-bot exhibits a good thermal deformation response with a bending angle ranging from 10° to 38° (Fig. 3B), such that its range of actuation is comparable to the un-skinned SMA skeleton (8°–41°). Although only up to five cycles are represented in Fig. 3, the mag-bots generally exhibited excellent repeatability. Indeed, many of the mag-bot designs were cycled over fifty times throughout the course of experiments, without detectable loss of bending angle recoverability. Nevertheless, microplasticity is well documented for NiTi SMAs undergoing thermomechanical cyclic loading^45–47^. As such, full-scale, sacrificial fatigue testing is perhaps warranted for prototyped designs of this mechanism when incorporated into applications.

As in the case of the uncoated SMA strips, there still exists upper and lower temperature thresholds above and below which actuation is unaffected, respectively. However, the full thermal transition zone is shifted to a higher temperature between roughly 40°–65° (as opposed to 25°–60° for the SMA alone), which is likely due to elastic resistance from the soft skin. To ensure that this widened temperature range does not significantly impact the latency of actuation, we also examine the deformation and temperature responses with respect to time (Fig. 3C,D). Figure 3C shows the response curve of three distinct mag-bot prototypes, each with an initial bending angle of 41° and skin thickness of 4 mm. For these experiments, magnetic induction heating occurs for 10 s, followed by 20 s of cooling, and each sample is tested for three consecutive cycles. Notably the presence of the elastic skin around the SMA sample restricts its deformation during heating but also creates an elastic restoring force that accelerate its recovery to the original state during cooling, hence the reduction in cooling time from 30 s (for the SMA skeleton alone) to 20 s (for the assembled mag-bot). Figure 3C demonstrates that significant actuation is preserved over the timescale of 10^1^ s, even in in the presence of the elastic skin. Furthermore, the deformation is nearly reversible from the first to second cycle, and fully reversible thereafter.

Another function of the elastic skin is its insulating effect. Figure 3D presents the time evolution of the mag-bot’s surface temperature during cyclic induction heating. We see that this temperature varies between 22 and 37 ℃, which is lower than that of the SMA strip alone (40–65 ℃). This is because induction heating only acts on the SMA, and not the external soft skin. As a result, the internal temperature of the mag-bot (Fig. 3B) exceeds the external temperature measured by infrared thermal imagery (Fig. 3D). Therefore, the external skin not only reduces the timescale of bending recovery during cooling, but also reduces the surface temperatures of the mag-bots to a safer range for human handling and interfacing with biological systems. A final and primary purpose of the external skin is to induce asymmetric surface interfaces and friction for locomotion.

### On the origins of asymmetric motion

**Figure 4:**
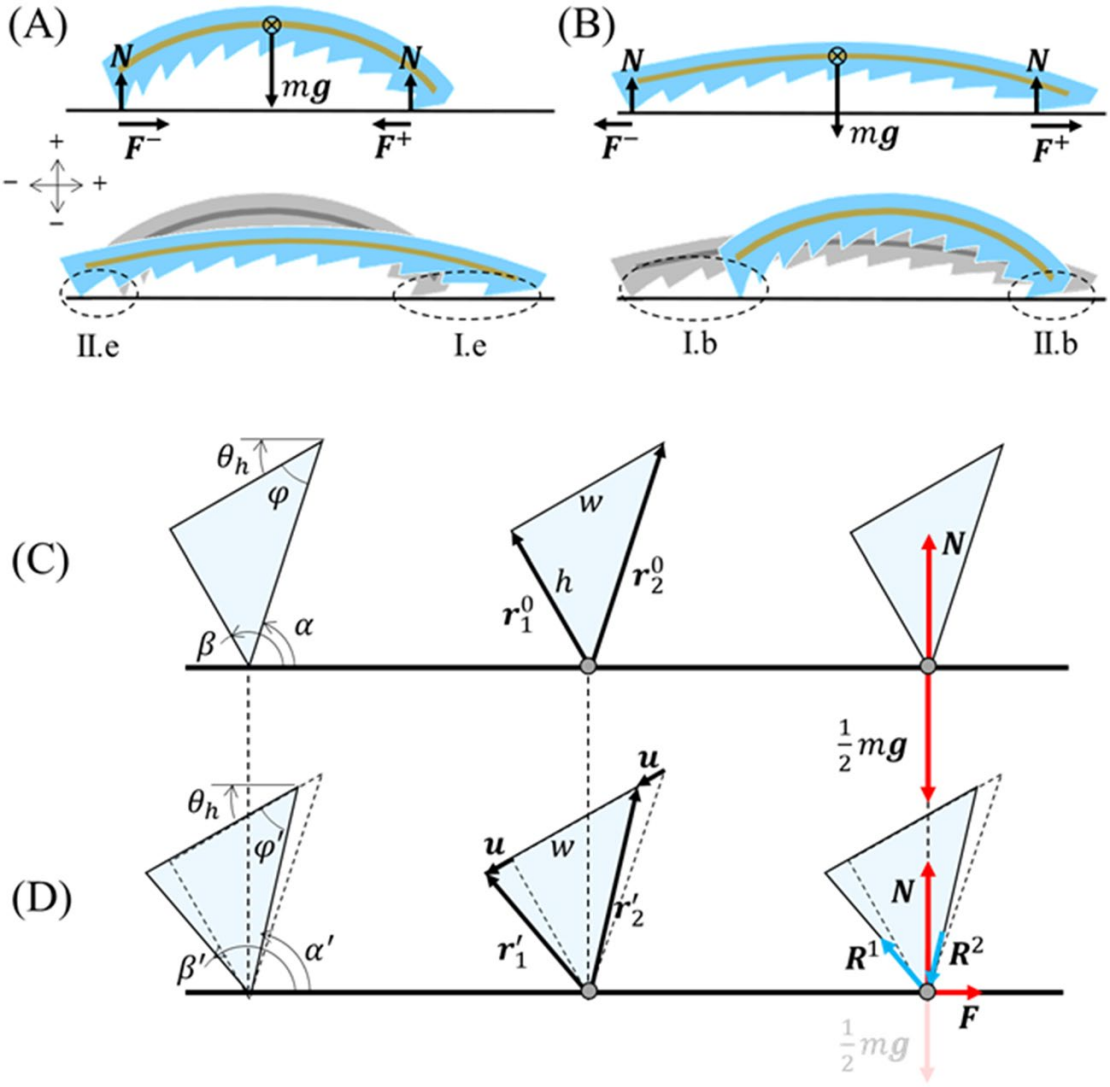
Analysis of asymmetric friction. Free body diagrams and designations of (**A**) Types II.e and I.e deformation for hindfoot and forefoot wedges during extension, respectively, as well as (**B**) Types I.b and II.b deformation for hindfoot and forefoot wedges during bending, respectively. (**C,D**) Close-up schematics of a hindfoot wedge undergoing Type II.e deformation (relative movement against the pattern during extension) is displayed (**C**) prior to deformation and (**B**) at the breaking of static friction. Leftmost schematics denote the angles pertinent to analysis, center schematics denote pertinent spatial vectors, and rightmost schematics display pertinent forces. For analogous schematics of Types I.e, I.b, and II.b deformation, see Fig. S4. Note that the direction of motion is along the positive horizontal axis (i.e., rightwards).

Unlike multimodal forms of peristaltic motion^13,17^, the first-harmonic actuation of the mag-bot is symmetric and may not power directional motion without a supplemental source of symmetry-breaking. Therefore, we design the mag-bot with an asymmetric surface pattern along its bottom face, consisting of a periodic set of right triangle-shaped, “sawtooth”-like wedges whose pattern angle, $\varphi$ (as measured between each wedge’s angled face and the local tangent to the SMA), may be adjusted to optimize movement (Fig. 4). While the geometry was patterned along the bottom of the mag-bot for ease of mold design and fabrication, only the frontmost and rearmost wedge geometries prove significant. This is because throughout actuation, the mag-bot only remains in roughly two-edge contact with the flat substrate it travels on. For convenience, we refer to the leading and tailing contact regions as the “forefoot” and “hindfoot”, respectively.

As reported by Tramsen et al., many factors influence the anisotropic friction of deformable “sawtooth” surface structures as they slide over surfaces, including both substrate and sawtooth properties (e.g., surface roughness, stiffness, geometry, etc.)^48,49^. However, here we assume that the coefficient of friction, $\mu$, is a constant material property governed only by the combination of substrate and silicon soft skin. Therefore, to understand what causes asymmetric motion, we need only consider the external forces acting on the mag-bot during one bending or actuation cycle (Fig. 4A,B), which are body force due to gravity ($W$), the normal force from the substrate at either foot ($N$), and the frictional forces acting on the forefoot (${F}^{+}$) and hindfoot (${F}^{-}$). From Fig. 4A,B, we see that the only external forces acting along the axis of motion of the mag-bot are the frictional forces at its two feet. The magnitudes of these frictional forces may be written as:

1 $$\left\{\begin{array}{c}{F}^{+}=\mu {N}^{+}\\ {F}^{-}=\mu {N}^{-}\end{array}\right.,$$

where ${N}^{+}$ and ${N}^{-}$ are the normal forces at the contact sites of the forefoot and hindfoot, respectively. For forwards motion to occur, one or both feet must undergo periods of both static friction (characterized by coefficient ${\mu }_{s}$) and kinetic friction (characterized by ${\mu }_{k}$). By definition, a foot begins sliding (i.e., undergoes the transition from static to kinetic friction) when $F/N$ first exceeds ${\mu }_{s}$. While neither foot is sliding, the mag-bot cannot undergo relative movement with respect to the substrate, and so one or both feet must break static friction. However, as revealed in the discussion below, simple equilibrium force analysis predicts that static friction does not break simultaneously for the two feet, due to their geometric asymmetry. Since static friction generally exceeds kinetic friction in magnitude, whichever foot breaks static friction first likely dictates the direction the mag-bot displaces towards. Specifically, if the forefoot breaks static friction first during extension or the hind foot breaks static friction first during bending, the magbot will move forwards. Furthermore, supposing static friction is broken by both feet, it becomes the difference between forefoot and hindfoot kinetic friction forces that governs the net displacement of the mag-bot. Through either effect, we may deduce the following scaling law:

2 $$\Delta x\sim {F}^{+}-{F}^{-}=\mu \left({N}^{+}-{N}^{-}\right),$$

where $\Delta x$ is the net horizontal mag-bot displacement in either phase of actuation.

This simple scaling intuitively suggests that whether it is due to the staggered breaking of static friction or an unbalanced force during simultaneous foot sliding, asymmetric mag-bot motion stems from the unequal distribution of normal forces between the fore and hindfoot. Therefore, we can reasonably predict the effects of foot geometry through equilibrium force analysis.

We now consider the forces (including internal reaction loads in the wedge) acting at the contact site for each of the feet during both extension and bending, independently. Adapting the terminology introduced by Tramsen et al. we distinguish two relative motion types for the sawtooth feet: Type I motion in which the tapered face of the pattern leads, and the relative movement of the substrate is “along” the pattern and Type II motion in which the contact edge leads, and the relative movement of the substrate is “against” the pattern. However, we also recognize that Type I and Type II motion differ between cases of extension and bending due to the bent conformation of the mag-bot. As such, we further denote deformation modes of the hindfoot and forefoot during extension as “Type II.e” and “Type I.e”, respectively, throughout this work (Fig. 4A). Similarly, deformation modes of the hindfoot and forefoot during bending are denoted as type ““Type I.b” and “Type II.b”, respectively (Fig. 4B). Sample schematics depicting the hindfoot wedge during Type II.e deformation are presented in Fig. 4C (before actuation begins) and Fig. 4D (at peak static friction equilibrium). For simplicity, we treat the solid wedge as a triangular element comprised of two linear springs (initially denoted by vectors ${\bf r}_{1}^{0}$ and ${\bf r}_{2}^{0}$, respectively—Fig. 4C) whose top ends are rigidly connected and separated by wedge width, $w$. The initial length of element one (the tailing element) is taken as the wedge height, $\left|{\bf r}_{1}^{0}\right|=h$, such that the length of element two (the leading element) is given by $\left|{\bf r}_{2}^{0}\right|=h/\text{sin}\varphi$. Note that while the pattern geometry is defined by angle $\varphi$, the initial local tangent angels of the mag-bot at the beginning of extension (${\theta }_{h}\approx 37.5^\circ$) and bending (${\theta }_{l}\approx 10^\circ$) (see Fig. 3B), also influence the element orientations with respect to the substrate. The orientations of the leading and tailing elements are defined by angles $\alpha$ and $\beta$, respectively (Fig. 4C,D).

Prior to deformation (Fig. 4C), the only forces acting on the edge are the locally distributed mag-bot weight, ${\bf W}^{\pm }=0.5\, m {\bf g}$, (where $m$ is mag-bot mass, $\bf g$ is acceleration due to gravity, and the weight is assumed evenly distributed between the feet given the approximate mass symmetry of the design) and resulting normal force, $\bf N$. However, upon actuation, the top of each element must move by some displacement $\bf u$ before static friction is broken. To simplify the model and based on experimental observations, we posit that static friction is broken while the norm of displacement is much smaller than the characteristic wedge size ($\left|{\mathbf{u}}\right|\ll w$). This allows us to treat $\bf u$ as roughly in-line with the initial mag-bot tangent angle (e.g. ${\theta }_{h}$ for Type I.e deformation), and as having the same magnitude at each element (see Fig. 4D, center). The tailing and leading elements may then be defined as ${\bf r}_{1}^{^{\prime}}={\bf r}_{1}^{0}+\bf u$ and ${\bf r}_{2}^{^{\prime}}={\bf r}_{2}^{0}+ \bf u$, respectively. Due to deformation of the wedge (e.g., the elongation of element one and compression of element two for Type I.e deformation), reaction loads ${\bf R}^{1}$ and ${\bf R}^{2}$ emerge and are counteracted in the horizontal direction by friction, $\bf F$, whose magnitude from Eq. (1) is $\mu N$ (see Fig. 4D, right). The vertical components of these loads are in equilibrium with the local weight, ${\bf W}=0.5\, m {\bf g}$ (taken as a constant) and the local normal force, $\bf N$, which may vary. The equilibrium conditions for the contact edge in the horizontal and vertical direction are respectively given by:

3 $$\left\{\begin{array}{l}\mu N+{R}^{1}\,\text{cos}\beta{^{\prime}}+{R}^{1}\,\text{cos}\alpha{^{\prime}}=0 \\ N-\frac{1}{2}mg+{R}^{1}\,\text{sin}\beta{^{\prime}}+{R}^{1}\,\text{sin}\alpha{^{\prime}}=0\end{array}\right.,$$

where angles $\beta{^{\prime}}$ and $\alpha{^{\prime}}$ are the angles of the first and second element with respect to the horizontal substrate at the static friction breaking conformation (e.g., ${\beta }^{^{\prime}}=\pi +{\theta }_{h}+{\text{tan}}^{-1}(u/h)$ and ${\alpha }^{^{\prime}}={\theta }_{h}+{\text{tan}}^{-1}\left[h/(w-u)\right]$ for Type I.e deformation). Sign convention is as indicated in Fig. 4A. Treating each element as a linear spring with spring constant $K=EA/\left|{\bf r}^{0}\right|$ (where $E$ is the material’s Young’s modulus, $\left|{\bf r}^{0}\right|$ is the initial element length, and area $A$ is treated as a fitting parameter), then the magnitude of reaction load in each element may be taken as $R=K\left(\left|{\bf r}^{^{\prime}}\right|-\left|{\bf r}^{0}\right|\right)$. Equation (3) host two unknowns: the displacement magnitude at which static friction is broken, $u$, and local normal force, $N$, which are solved for numerically. Parametric values used in this study are presented in Table 1. For the full set of derived angle relations used for $\alpha{^{\prime}}$ and $\beta{^{\prime}}$ for each type of deformation, see the “Materials and methods” section and Fig. S4.

**Table 1:** Model parameters.

Symbol	Definition	Value	Basis
$\mu$	Coefficient of static friction	$0.2$	Free parameter ($0<\mu \le 1$)
$A$	Element cross-sectional area	$4.4\times {10}^{-8} \,{\text{m}}^{2}$	Fitting parameter
$E$	Young’s modulus	$69 \,\text{kPa}$	Material spec
$m$	Mag-bot mass	$6.1\times {10}^{-4}\, \text{kg}$	Measured avg
$w$	Mag-bot wedge dimension	$3.8 \times {10}^{-3}\, \text{m}$	Measured
$g$	Gravitational constant	$9.81\, \text{m }{\text{s}}^{-1}$	Constant

**Figure 5:**
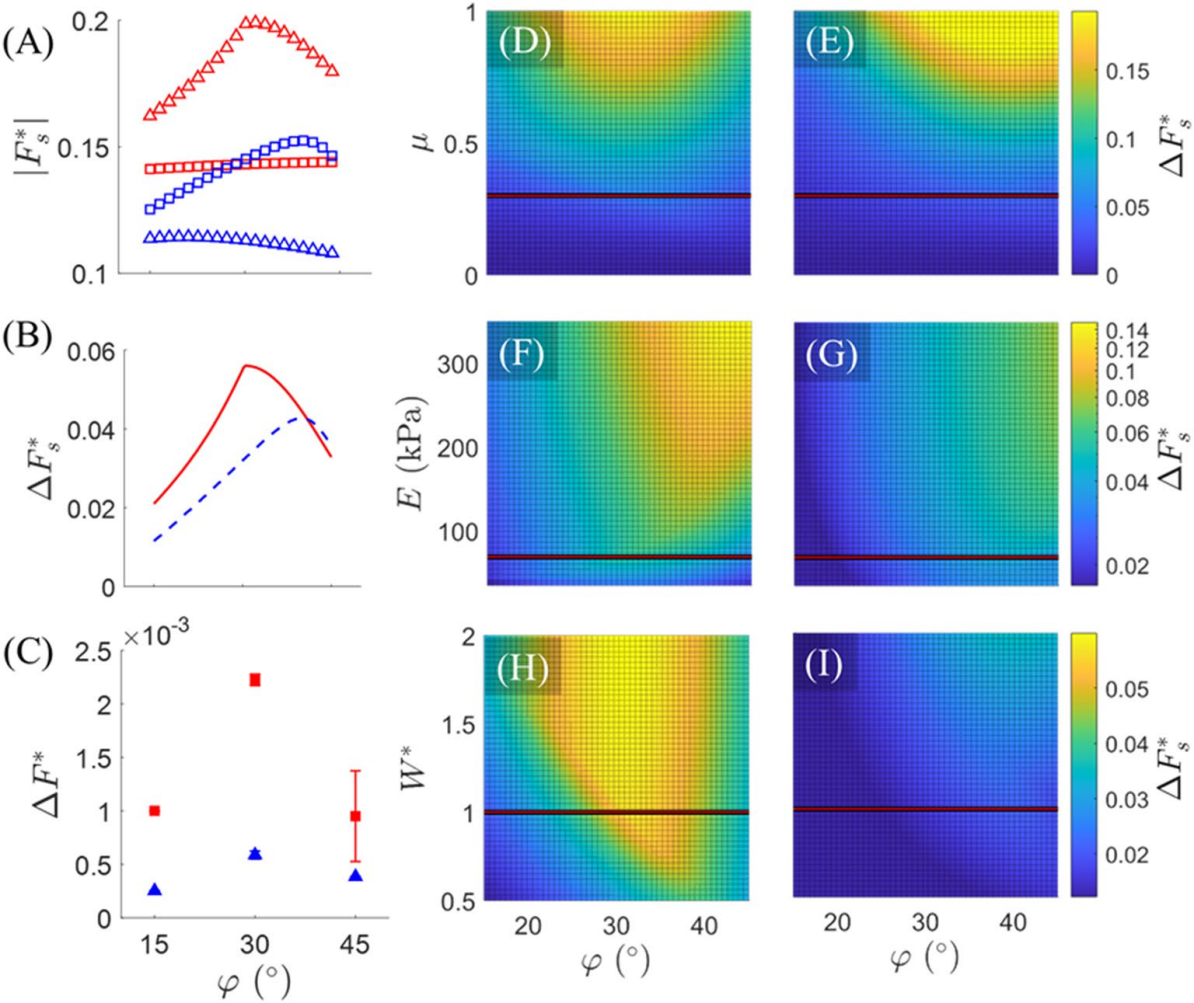
Model results. (**A**) The magnitude of static friction force (normalized by mag-bot weight), as predicted by the model, is plotted with respect to $\varphi$ for Type I.e (red squares), Type II.e (red triangles), Type I.b (blue triangles), and Type II.b (blue squares) deformation types. (**B**) The difference in forefoot (squares) and hindfoot (triangles) static friction (normalized by mag-bot weight), as predicted by the model, for both extension (solid red line) and bending (dashed blue line) is plotted with respect to $\varphi$. (**C**) The experimentally estimated mean unbalanced force during both extension (red squares) and bending (blue triangles), is plotted with respect to $\varphi$. (**D,E**) Model-predicted values of normalized net friction ($\Delta {F}_{s}^{*}=({F}^{+}-{F}^{-})/\text{mg}$) with respect to $\varphi$ and (**D,E**) friction coefficient ($\mu$), (**F,G**) skin modulus ($E$), and (**H,I**) normalized weight (${W}^{*}=m/[6.1\times {10}^{-4}\, \text{kg}]$). (**D,F,H**) Represent extension, while (**E,G,I**) represent bending. (**D–I**) Horizontal lines represent the parametric slice presented in (**B**) (for which $\mu =0.3$, $E=69 \,\text{kPa}$, and $m=6.1\times {10}^{4} \,\text{kg}$, the latter two of which are based on the true mag-bot parameters).

Figure 5A displays the magnitude of each foot’s normalized static friction force ($\left|{\bf F}_{s}^{*}\right|=\left|{\mathbf{F}}\right|/{mg}$) with respect to pattern angle as predicted by the model for all four types of foot deformation. During both extension and bending, the model predicts that Type II deformation (red triangles or blue squares) against the pattern angle begets higher frictional forces than Type I deformation (red squares or blue triangles). Consequently, the net difference between forefoot and hindfoot friction ($\Delta {F}_{s}^{*}=({F}^{+}-{F}^{-})/\text{mg}$), displayed in Fig. 5B for both extension and bending, always yields positive (i.e., forwards) force, thereby implicating forwards motion, which is consistent with experimental observations. Interestingly, $\Delta {F}_{s}^{*}$ predicted by the model displays a biphasic relationship with respect to $\varphi$, during both extension and bending phases of actuation. This biphasic relation is qualitatively consistent with the experimentally estimated net unbalanced force, $\Delta {F}^{*}$ presented in Fig. 5C (see “Materials and methods” section for characterization details). Notably, Fig. 5A,B represent the estimated static friction forces necessary to induce sliding of each foot, and the net static frictional force between feet, respectively, whereas Fig. 5C captures the lumped effects of static and kinetic frictional force differences throughout each mode of deformation (hence omission of the subscript “s” in Fig. 5C). This likely explains why the quantities of Fig. 5C are an order of magnitude smaller than those of Fig. 5B and direct quantitative comparison is not intended. Nevertheless, the model capably reproduces the same biphasic relation.

Element area $A$ was set as a free parameter to demonstrate that the model can predict emergence of an optimal pattern angle between $\varphi =30^\circ$ and $45^\circ$ as seen experimentally. The model indicates that the emergence of this biphasic net friction with respect to $\varphi$ results from comparably biphasic $|{\bf F}_{s}^{*}|-\varphi$ relations for Type II modes of deformation, “against” the pattern angle (Fig. 5A, red triangles and blue squares). This biphasic behavior likely stems from the relative orientation of the sloped face with respect to the substrate, $\alpha{^{\prime}}$ (Fig. 5D). For example, in the case of Type II.e motion (Fig. 4C,D) if ${\alpha }^{^{\prime}}$ (the sum of the pattern angle, $\varphi{^{\prime}}$, and tangent angle, ${\theta }_{h}$) exceeds $90^\circ$ at any point during the deformation, the reaction load, ${R}_{2}$, would go into tension (as opposed to compression, as drawn), which would reduce the normal force and (therefore) static friction. This is most likely to occur at higher pattern angles such as $45^\circ$. Meanwhile, low pattern angles such as $15^\circ$ will reduce the vertical component of ${R}_{2}$, thereby also reducing the magnitude of normal force and (therefore) static friction. Thus, there exists some optimal angle between these ranges that maximizes friction force. In contrast, the effects of $\varphi$ on friction for Type I deformation modes “along” the pattern angle (Fig. 5A, red squares and blue triangles) are monotonic and far less pronounced. In fact, the model predicts that a higher pattern angle will minutely increase forefoot friction during extension, whereas it will decrease hindfoot friction during bending. In both cases, this suggests that a shallower angle between the leading face and substrate culminates in a higher friction force for Type I motion.

Notably, the biphasic relation persists for a wide range of friction coefficients (Fig. 5D,E), skin moduli (Fig. 5F,G), and mag-bot weights (Fig. 5H,I). Furthermore, increasing $\mu$, ${W}^{*}$, and $E$ (considering no effect that this might have on ${\theta }_{h}$ or ${\theta }_{l}$) predicts an increase in the degree of frictional difference between the forefoot and hind foot. That $\Delta {F}_{s}^{*}$ scales directly with all three parameters suggests that the greater the order of magnitude of friction forces, the greater the difference in the forces at which static friction is broken. However, this is not necessarily indicative of better performance of the mag-bots. As discussed later in the context of rough surfaces, there appear to be high-friction conditions under which the mag-bots cannot break static friction at either foot or higher values of kinetic friction hinder net displacement and cause repeated “sticking” (i.e., transition back into static friction) during actuation. Generally, since this model does not estimate frictional forces during kinetic friction, it cannot be used to quantitatively predict exact net displacements of the mag-bots. Rather, it is used to explain the cause of anisotropic friction, elucidate the likely cause of an optimal foot angle, and help qualitatively explain observed trends in the mag-bots performance, as detailed in the following sections.

### Quantifying mag-bot motion

**Figure 6:**
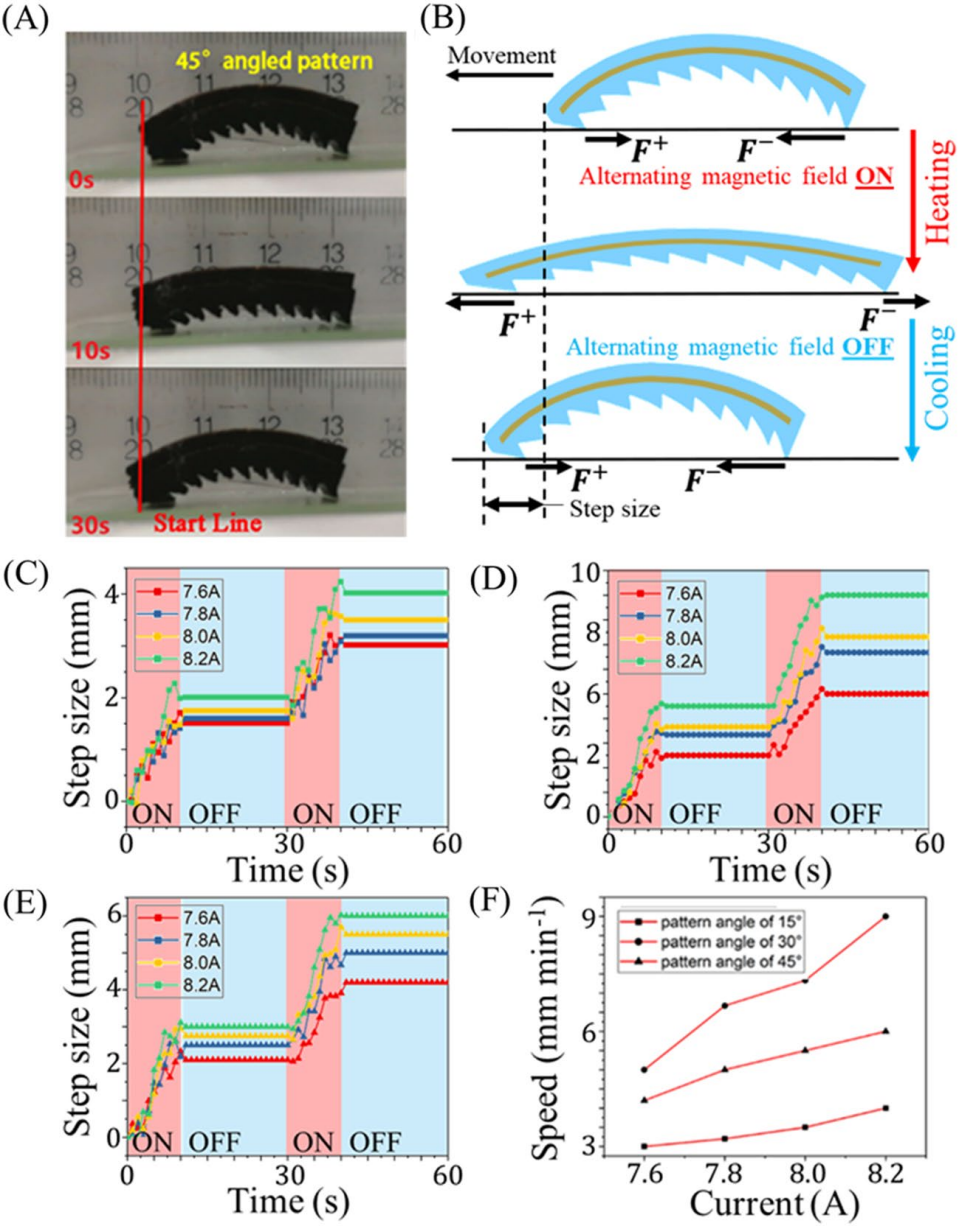
Motion principle and kinematic behavior analysis of mag-bot. (**A**) Snapshots of a mag-bot undergoing one thermal load cycle display movement leftwards. The top snapshot is presented at the beginning of heating (0 s); the middle snapshot is shown at the end of heating and beginning of cooling (10 s); and the bottom snapshot depicts the end of cooling (30 s). (**B**) A schematic illustrates the direction of frictional forces on the forefoot (${F}^{+}$) and hindfoot (${F}^{-}$) as well as the emergent step size after one loading cycle (not to scale). (**C–E**) Step sizes are plotted with respect to time for mag-bots with **(C)** 15°, **(D)** 30°, and **(E)** 45° pattern angles for two cycles of thermal loading, over a range of magnetically induced currents ($I\in \left[\text{7.6,8.2}\right]\, \text{A}$). **(F)** Velocity is plotted with respect to induction current for mag-bots with pattern angles of 15° (squares), 30° (circles), and 45° (triangles).

We here quantify mag-bots’ movements and speeds as they are subjected to multiple actuation-recovery cycles on a level, rigid, glass substrate. Figure 6A depicts snapshots of a mag-bot with a pattern angle of 45° as it undergoes a single loading cycle and moves leftwards. For additional visualization, see Fig. S5 and Movie S1. Starting from a bent state (0 s), the alternating magnetic field is turned on to induce extensile actuation of the mag-bot (10 s). Ceasing magnetic actuation at 10 s induces a cooling of the material, causing the mag-bot to bend back to its original state and marking the beginning of the next cycle. As expected, the existence of asymmetric patterns on the surface of the mag-bot induces directional motion (Fig. 6B). During the extension phase, the forefoot friction is oriented backwards (i.e., opposes net forward movement), and the hindfoot friction is forwards (i.e., encourages net forward movement). However, the forefoot’s static friction-breaking force is smaller than that of the hindfoot during extension, as evidenced by a finite translation ${\Delta}_{e}$ of the of the mag-bot leftwards (i.e., in the intended direction of motion). By contrast, during the recovery phase, the forefoot’s friction is forward motion), while the hindfoot’s friction is backward. Yet, as during heating, the difference in frictional forces during cooling induces a forward translation with step size ${\Delta}_{r}$. Together, a full cycle therefore yields a combined step size of ${\Delta}={\Delta}_{e}+{\Delta}_{r}$ and the velocity of a mag-bot is defined as $v=\Delta /T$ where $T$ is the time of a full cycle.

Figure 6C–E show experimental results of the maximum step size of a mag-bot for various pattern angles (15°, 30° and 45°, respectively) over a range of different induction currents (correlating directly to the magnetic field strength). The corresponding velocities for mag-bots with all three pattern angles are plotted with respect to the induction current (and therefore strength of the magnetic field) in Fig. 6F. Regardless of pattern angle or current the forefoot universally displaces forwards during the heating phase and forwards movement is always initiated within the first 2.5 s of actuation indicating that static friction is broken relatively quickly for the Type I.e deformation mode. Additionally, very little backwards regression of the forefoot occurs during any of the observed cooling phases suggesting that the forefoot generally remains in static friction during the Type II.e deformation mode.

As expected, increasing field strength increased the rate at which the mag-bots were heated, thereby increasing their effective step size for the selected heating duration (10 s). That the step size was not identical for every current indicates that the upper temperature threshold of ~ 65 °C (discussed in reference to Fig. 3B) was not ubiquitously reached by the mag-bots upon induction cessation. The existence of an upper limit (above which further heating induces no further extension) suggests that for a given current, there is a maximum heating time and step size that will produce usable extension. For the case of the 8.2 A current, a heating duration of 10 s was found necessary to reach the lower tangent angle threshold on the order of 10$^\circ$ (corresponding to the upper temperature limit) (see Fig. 3D). Regardless of improved heating time (which could be comparably improved by increasing the number of induction coils or alternating current frequency) the limiter of mag-bot speed resides in its cooling duration which accounts for 67% of the best cycle times observed. Thus, the extent to which walking speed could be improved by reduced heating latency is limited to less than 33%.

### Effects of the elastomeric skin parameters

**Figure 7:**
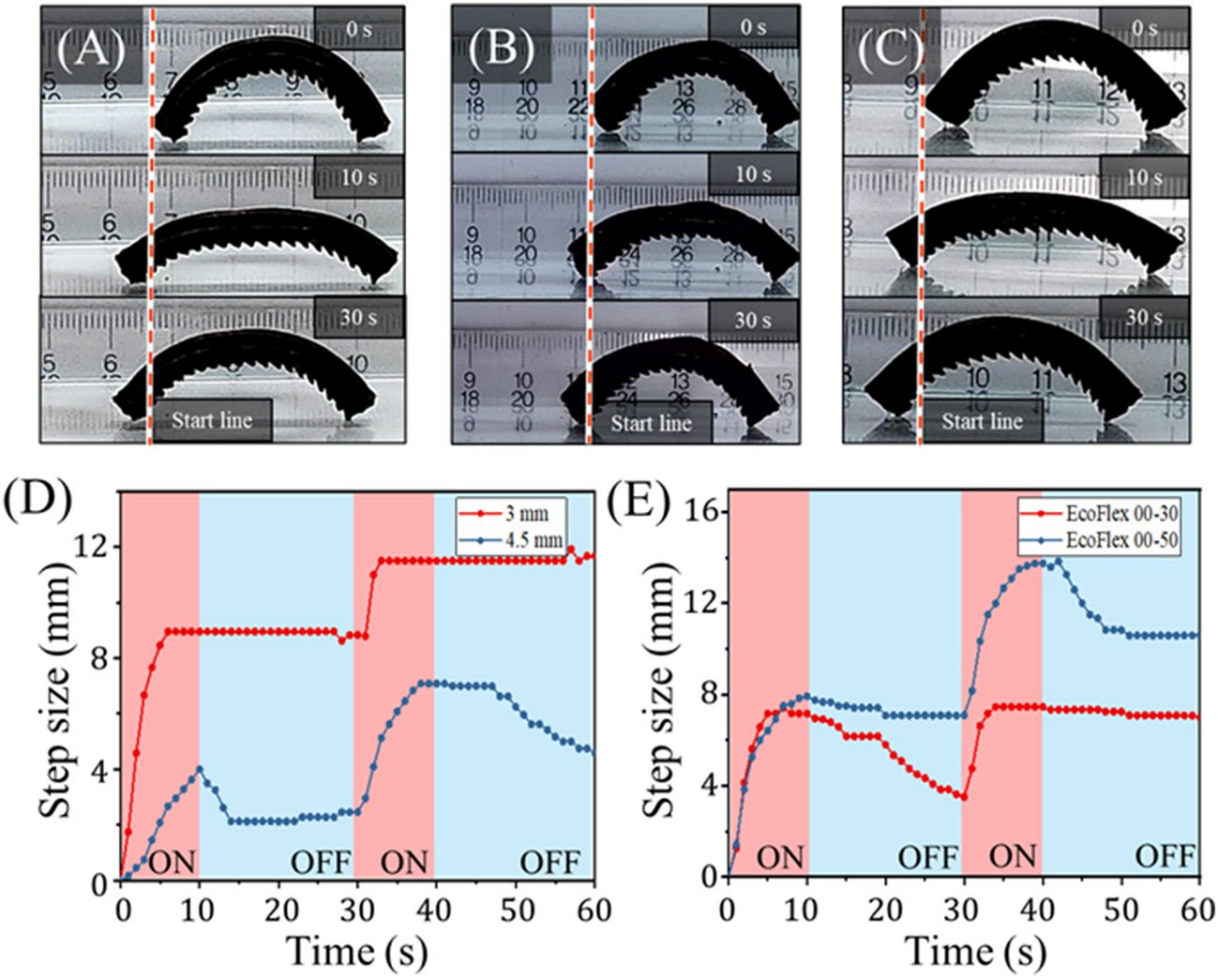
Analysis of mag-bot with different skin parameters. (**A–C**) Snapshots of mag-bots with 30° pattern angles are shown at (top) 0 s (before actuation), (middle) 10 s (at the end of one extension cycle), and (bottom) 30 s (at the end of one complete cycle). The mag-bot in (**A**) has 4.5 mm-thick skin comprised of EcoFlex 00-30 for comparison to the control design (3 mm-thick skin comprised of EcoFlex 00-30). The mag-bot in (**B**) has 4 mm-thick EcoFlex 00-30 skin, and the mag-bot in (**C**) has 4 mm-thick EcoFlex 00-50 skin. (**D**) Net forefoot displacement is plotted with respect to time for the control magbot from Fig. 6A, and mag-bot of (**A**). (**E**) Net forefoot displacement is plotted with respect to time for the mag-bots of (**B,C**).

In addition to varying the pattern angle and magnetic field strength, we also investigated the effects of varying elastomeric skin thickness (Fig. 7A–C) and modulus (Fig. 7D,E). So as not to alter the patterned feet, skin thickness was measured and varied only from the top face of the SMA skeleton to the top face of the elastomer. Increasing the skin thickness from 3 to 4.5 mm greatly reduced the mag-bot’s extension length, and therefore step size during the heating phase (Fig. 7C). Furthermore, it appears the forefoot consistently slipped during the cooling phase as indicated by the decrease of the blue curve from 10–30 to 40–60 s in Fig. 7D. These effects greatly reduced performance and speed, and they are attributed to the higher bending stiffness associated with increasing the mag-bot skin’s cross-sectional area. Thus, in designing these mag-bots for applications, the skin thickness should be selected considering the trade-off between thermoresponsive extensibility, and the desired degree of thermal insulation.

Elastomer hardness was varied by using two different materials: EcoFlex 00-30 (the control used throughout experiments) and EcoFlex 00-50. The Young’s moduli of these materials (taken as the tangent moduli at low strain per Fig. S6) were measured as approximately $70\, \text{kPa}$ for EcoFlex 00-30, and $330\, \text{kPa}$ for EcoFlex 00-50. The model predicts that an increase in modulus from $70$ to $330$ kPa would result in an approximately four-fold increase in the static friction threshold between the hindfoot and forefoot (Fig. 5E,F). Indeed, the mag-bot with the EcoFlex 00-50 skin does exhibit a roughly 50% greater net step size after two cycles over its EcoFlex 00-30 counterpart (Fig. 7E). Notably, both mag-bots’ forefeet slip during the cooling phase of one of the two cycles, although these mag-bots were both tested with 4 mm-thick skins, and so this seemingly stochastic effect may be mitigated by using a thinner skin (e.g., 3 mm). Nevertheless, these results suggests that skin modulus is an impactful parameter and should be considered for purposeful design.

### Movement characterization on rough terrain

**Figure 8:**
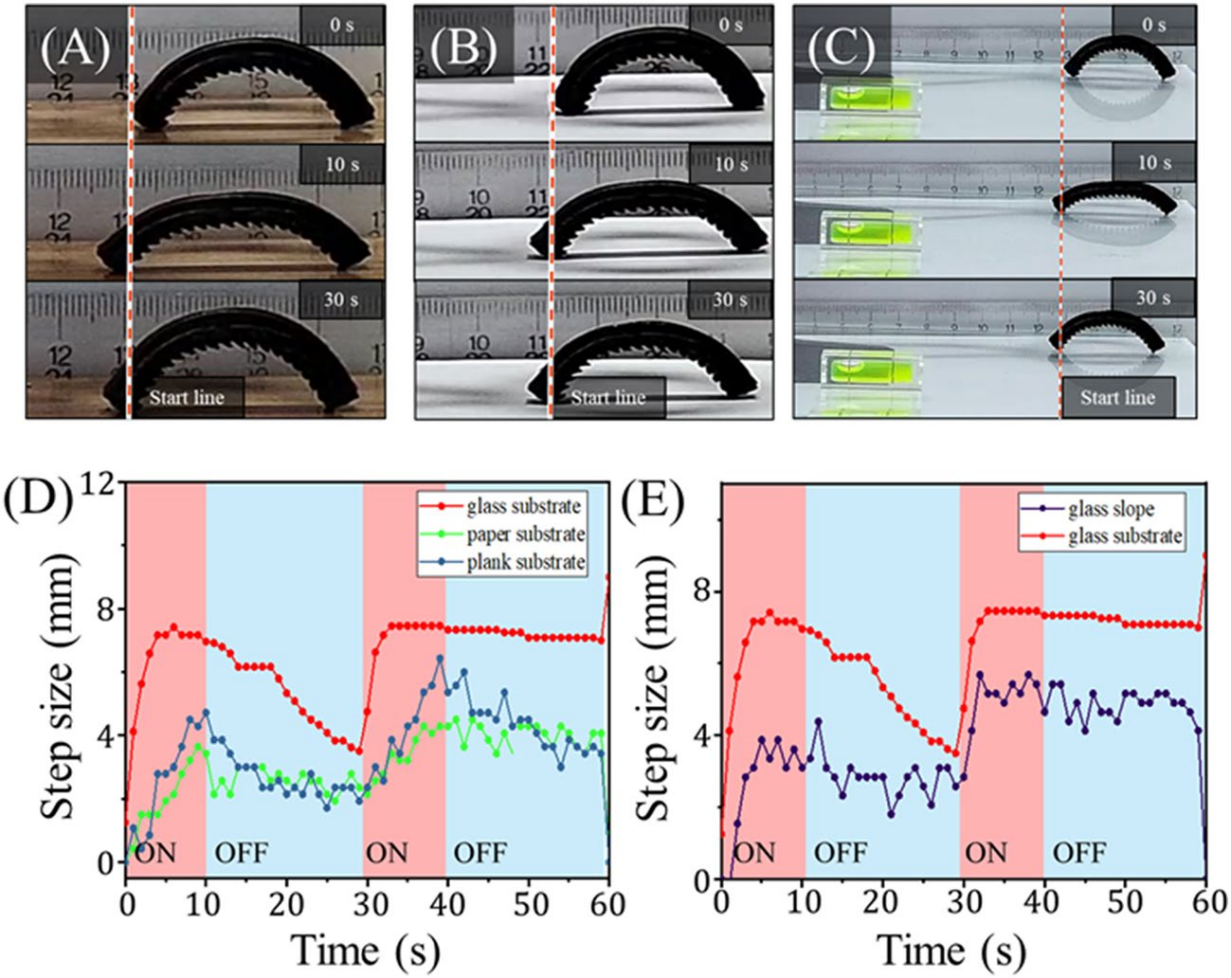
Analysis of mag-bots on rough terrain. (**A–C**) Snapshots of mag-bots with 30° pattern angles are shown at (top) 0 s (before actuation), (middle) 10 s (at the end of one extension cycle), and (bottom) 30 s (at the end of one complete cycle). The mag-bot is depicted walking on **(A)** a wooden plank, **(B)** printer paper, and **(C)** up a 5$^\circ$ inclined glass slope (see the level at the bottom left). **(D)** Net forefoot displacement is plotted with respect to time for the mag-bot walking on wood, paper, and the control condition of a level glass substrate. **(E)** Net forefoot displacement is plotted with respect to time for the mag-bot walking up the glass slope and the control condition (i.e., level glass).

To characterize the robustness of the mag-bots’ locomotion, we tested the design with 4 mm-thick, EcoFlex 00-30 skin and a $30^\circ$ pattern angle on a variety of substrates. The results of the mag-bot walking on wood, paper, and up a $5^\circ$ glass slope are presented in Fig. 8. While the mag-bot proves capable of walking on all three substrates, its performance is decreased as compared to the control condition (i.e., a level glass substrate). Furthermore, the forefoot displacement exhibits a large degree of noise, likely indicating that the mag-bots were repeatedly breaking and re-entering static friction with these substrates. The decreased performance of the mag-bots on paper and wood is attributed to an increase in the frictional coefficient. While the model predicts an increased difference between forefoot and hindfoot static friction thresholds ($\Delta {F}_{s}^{*}$) at higher values of $\mu$, this corroborates that $\Delta {F}_{s}^{*}$ does not scale directly with performance, as one must also consider whether static friction can be broken by either foot and how kinetic friction influences performance.

To probe the limits of static friction breaking, mag-bots were also tested on tile, cement, and concrete surfaces whose defects’ length scales are on the same order of magnitude as that of the patterned feet ($\sim {10}^{-4}{-}{10}^{-3}$ m). Under such conditions, neither foot breaks static friction and the mag-bots do not move. That neither foot breaks static friction implicates actuation force as a limiting factor for these mag-bots on surfaces with high friction coefficients. To mitigate this effect, heavier mag-bots operating on rough surfaces may be built with wider SMA strips to improve actuation force. Alternatively, weight and elastic resistance (from the elastomeric skin) may be reduced through some combination of decreased skin thickness and modulus. Additionally, to help the mag-bots circumvent or overcome obstacles and rough surfaces in applications, their elastomeric skins have been embedded with Fe_3_O_4_ nanoparticles such that they may be manipulated or reoriented by permanent magnets as a contingent resort (Fig. S7). Notably, test confirm that the inclusion of these magnetic particles neither pulls the mag-bots towards the induction coils’ alternating magnetic fields (Movie S2), nor prohibits the mag-bots from moving away from them (Movie S3), which together ensure that it is the SMA actuation and foot pattern that induce motion.

To probe the lower limit of frictional coefficients, the mag-bots were also tested on glass slides wetted with water. However, under such conditions, both feet of the mag-bot broke static friction almost immediately upon actuation, culminating in zero net displacement. This is consistent with the expectations of the model (Fig. 5D,E), which predicts that when $\mu$ is sufficiently low $\Delta {F}_{s}^{*}$ is negligible. Together, these findings indicate that there exists some optimal friction coefficient for mag-bot performance, which should be characterized specifically for a given design and application.

### Cargo carrying by an unbound, soft mag-bot

**Figure 9:**
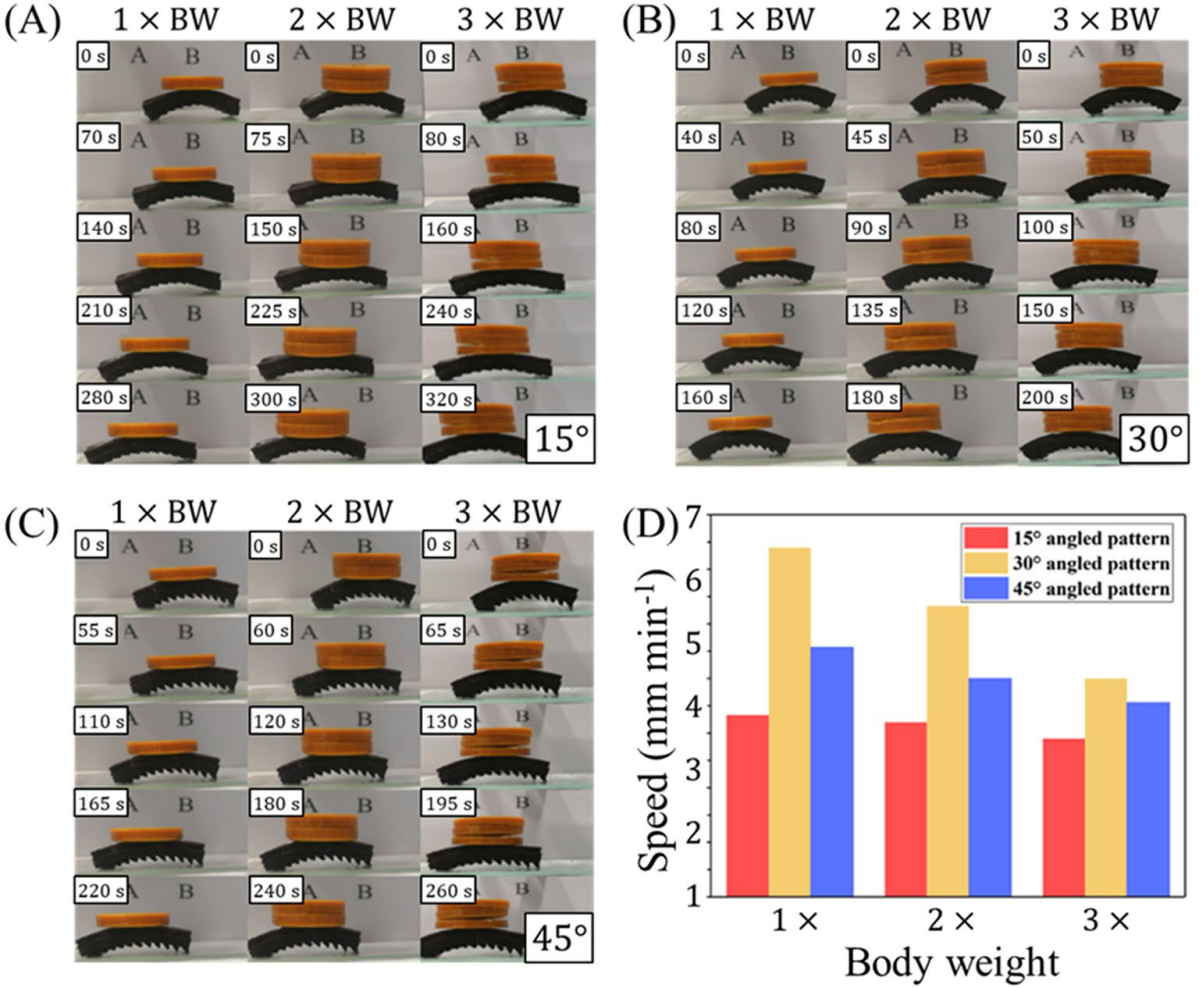
Load motion experiment of mag-bot with different angled pattern. **(A–C)** Mag-bots with **(A)** 15°, **(B)** 30°, and **(C)** 45° are displayed, each undergoing one cycle of loading (chronologically from top-to-bottom) while carrying loads that are (left) 1$\times$, (center) 2$\times$, and (right) 3$\times$ their body weight. **(D)** Travel speeds of mag-bots with different pattern angles of (red) 15°, (yellow) 30°, and (blue) 45° under loads of (left) 1$\times$, (center) 2$\times$, and (right) 3$\times$ a mag-bot’s body weight are displayed.

In addition to characterizing the movement of unburdened mag-bots, we also assess their efficiency while carrying external loads. For this, we carried out experiments in which cargo was placed centrally on top of the mag-bots (Fig. 9A–C, Movies S4–S6). Cargo loads of 1$\times$, 2$\times$ and 3$\times$ a mag-bot’s body weight were studied. Motion efficiency under these three loads for the three pattern angles (15°, 30° and 45°), are reported in Fig. 9D. The full set of measured travel speeds and efficiencies (with respect to unloaded speed) for mag-bots carrying 0$\times$, 1$\times$, 2$\times$, and 3$\times$ their own BW are reported in Table 2.

**Table 2:** Speed of mag-bots under various loading conditions using an induction current of 8.2 A.

Cargo load ($\times$ BW)	Speed (mm/min) (% Efficiency = % Unloaded speed/Loaded speed)		
	15° angled pattern	30° angled pattern	45° angled pattern
0$\times$	4.00 (100%)	9.00 (100%)	6.00 (100%)
1$\times$	3.33 (83%)	6.40 (71%)	4.57 (76%)
2$\times$	3.20 (80%)	5.33 (59%)	4.00 (67%)
3$\times$	2.90 (73%)	4.57 (51%)	3.56 (59%)

Parameters were conserved throughout experiments, unless specified otherwise (e.g., as in the cases of pattern angle, induction current, and cargo weight). The induction current was maintained at 8.2 A and the mag-bot skin was comprised of 4 mm-thick EcoFlex 00-30.

Generally, these results show that the speed of a mag-bot is only moderately affected by cargo whose weight is on the order of the mag-bot’s body weight. The trend in speed reduction is consistent across all three pattern angles and the intermediate angle (30°) still yields the fastest motion in all cases while the lowest angle (15°) yields the slowest motion. The loss in speed remains less than 50%, even when the cargo load is three times the mag-bot’s body weight. This indicates that, at least for mag-bots fabricated at this length scale, their specific weight-carrying capacity renders them eligible for cargo transport applications.

## Conclusions

This study leverages the first principles of soft actuation and anisotropic geometries that are regularly coupled in nature to induced peristaltic motion. While living organisms such as fly larvae utilize multimodal deformations and microscopic surface patterns to induce highly ordered locomotion^17^, we here distilled these mechanisms into perhaps their simplest analogues: namely, harmonic, unimodal (bending) actuation and a macroscopic wedge-shaped interface geometry. Thus we designed a set of maggot-inspired robots that achieve comparable, unidirectional locomotion without the need for fully peristaltic actuation. Their actuation is achieved by a strip of SMA that respectively heats and cools when introduced or removed from an alternating magnetic field, while the anisotropic geometry is introduced by a soft skin that envelopes the SMA strip, and which serves the secondary purposes of thermal insulation for safer handling and elastic storage for enhanced actuation recovery. The simplicity of this design is advantageous in a few ways. For instance, this mag-bot is—to our knowledge—but one of a few such soft robots that display unconfined, single-direction, peristaltic-like motion while carrying load in the absence of a tethered power supply. This is made possible by not only the simplicity of its design (i.e., lack of complex, local mechanisms), but also the simplicity of an orientation-independent, alternating magnetic field as its stimulus. While comparably simple, magnetically driven designs such as those introduced by Hu et al.^20^ or Lu et al.^35^ demonstrate remarkably robust locomotion, our design’s utilization of SMA is unique in that it requires no specific path or sense for its actuating magnetic field and merely requires that the field alternate sufficiently to induce heating. Second, the simplicity of this mag-bot design renders it inherently scalable. The design model introduced here is not inertial, and therefore remains theoretically applicable at smaller length-scales in dry environments. Furthermore, the magobts’ minimalist compositions (i.e., an SMA strip embedded in a soft skin) could be feasibly fabricated at smaller length scales using scalable molding methods or 3D printing. Additionally, the mag-bots examined here displayed relatively high specific-weight carrying capacities that will likely only improve in applications where they are scaled downwards in size, as governed by square-cube scaling laws for structural members.

Nevertheless, these mag-bots are not without limitations. Most notably, the timescale of actuation is on the order of 10^1^ s, while the length scale of travel is on the order of 10^–3^ m such that the normalized travel speeds are on the order 10^–2^ body lengths per minute. Furthermore, the current study only examines the movement of these mag-bots on level substrates, and early proof-of-concept work in their motility up inclines indicates a further reduction in movement speed. As such, future work will focus on improving the travel speed of mag-bots in the absence of load. In any case, the minimalism of these mag-bot’s design and fabrication strengthens their potential to be scaled downwards in size. This scalability, combined with the advantages of flexibility, untethered motion, and high specific load-carrying capacity exemplified in this work render this mag-bot promising for applications in which soft-body transport must occur through small spaces or with cargo in tow. Furthermore, with a portable alternating magnetic field source or suitably developed induction coil infrastructure (e.g., a magnetic resonance imager or pipe enveloped by periodic Helmholtz coils), these mag-bots could be made to operate over longer relative traversal distances. Prospective applications include monitoring of pipelines for damage detection, geological exploration, or remote small object recovery from confined spaces.

## Materials and methods

### Fabrication of the mag-bots

The fabrication process of the mag-bot is shown in Fig. S1. A thin rectangular specimen (40 mm $\times$ 4 mm $\times$ 0.1 mm) of NiTi shape-memory alloy (0.8 wt% Si, 43.8 wt% Ti, 55.4 wt% Ni, see Fig. S2) was fabricated using a laser cutter (Eagle, X-6060). An A-type silica gel (EcoFlex 00-30, Smooth-on) and B silica gel were mixed with a 1:1 mass ratio, and magnetic Fe_3_O_4_ nanoparticles with a 10% mass fraction were added. While the inclusion of Fe_3_O_4_ nanoparticles were found not to significantly influence magneto-thermal heating, they enable the mag-bot to be manipulated using a permanent magnet (Fig. S7), which provides users a means of reorienting the mag-bots and perhaps helping them overcome obstacles without direct access, albeit at a relatively short range. Thorough mixing was conducted using a magnetic stirrer for 10 min. The mixture was then poured into rectangular molds housing the SMA skeletons and containing different pattern angles of 15°, 30° and 45°. The molds were allowed to sit for 20 min to ensure that bubbles in the material could completely escape. They were then placed in a vacuum drying box at 80 °C for 4 h. Once the molds were removed, they were allowed to cool to room temperature. Ultimately, three different prototypes of mag-bots with surface pattern angles of 15°, 30° and 45° were fabricated with masses of 0.6040 g, 0.6000 g, and 0.6845 g, respectively. All mag-bots’ dimensions were approximately 42 mm $\times$ 6 mm $\times$ 4 mm, unless specified otherwise.

### Control of magnetothermal deformation

The actuation of both the SMA skeletons and fully fabricated mag-bots were independently assessed during thermal loading using the experimental set up depicted in Figs. 2A and 3A, respectively. The magnetic coil was placed a height of approximately 40 mm above the mag-bots and oriented with its central axis vertical during testing. The coil had 2 turns with a pitch of 6 mm, an internal diameter of 32 mm, and external diameter of 42 mm. The magnetic field was generated using an alternating current of 8.2 A at an alternating frequency of 425 kHz. When positioned directly under the central axis of the coil, the mag-bots could traverse on the order of 15 mm while the coil remained stationary (Movie S3) before no longer actuating sufficiently to locomote. Therefore, the magnetic coil was generally positioned to remain within 40 mm of the mag-bot throughout experiments. Temperature was increased from 20 to 80 ℃ in increments of 5 ℃ and the equilibrium deformation, as characterized by the bending angle, was recorded at each temperature. Surface temperature readings were measured by an infrared imaging system, FLIR T540. The total time of each cycle (40 s for the SMA and 30 s for the mag-bots) was set based on the observed actuation cycle time. Actuation time (10 s) was taken as the time needed for the sample to reach its bent, equilibrium state upon magnetic induction heating. Similarly, restoration time (30 s for the SMA and 20 s for the mag-bot) was taken as the time needed for the specimen to recover to its initial shape during ambient cooling. Notably, the restoration times differed between the independent SMA skeleton and integrated mag-bots due to elastic restoring forces imparted by the mag-bots’ soft skins. To obtain statistically relevant measurements, these tests were performed on three different samples where each sample was tested three times. To report the time-dependency of actuation and temperature, measurements were taken with a frequency of 1 Hz.

### Testing the mag-bots

The mag-bots were placed onto level glass substrates one at a time and exposed to the 30 s magnetic induction cycles as described above. Their travel speeds were recorded over several actuation cycles and taken as the step size (defined by Fig. 5B) per unit time. Speeds were measured under various induction currents (ranging from 7.6 A, 7.8 A, 8.0 A, and 8.2 A at an alternating frequency of 425 kHz) and pattern angle angles (of 15°, 30°, and 45°). The speeds of the mag-bots were measured both without (Fig. S2, Movie S1) and with (Movies S4–S6) cargo loads as depicted in Figs. 6A and 9A–C, respectively. For the loaded conditions, we consistently used an induction current of 8.2 A. The masses used (0.6138 g, 0.6129 g, and 0.6151 g) were each on the order of one mag-bot body weight (BW) and were placed centrally on top of the mag-bots. The method of graphical speed measurement is illustrated through Fig. S5.

### Experimentally characterizing anisotropic friction

To estimate the normalized unbalanced friction force, ${\Delta}{F}^{*}\approx ({F}^{+}-{F}^{-})/\text{mg}$, we simply tracked the geometric centers of the mag-bots within distinct frames of video footage. Their average accelerations along the horizontal axis during bending and extension were coarsely estimated using finite differences in position ($\Delta x$) and time ($\Delta t$) between frames as $\overline{a }\approx\Delta x/\Delta {t}^{2}$. Considering the forces acting on the mag-bot (Fig. 4A,B), the net unbalanced force along the horizontal axis can be written as:

4 $$\Delta F=ma\approx m\frac{\Delta x}{\Delta {t}^{2}},$$

from which the normalized force may be written:

5 $$\Delta {F}^{*}\approx {g}^{-1}\frac{\Delta x}{\Delta {t}^{2}}.$$

Equation (5) was used to estimate the values reported in Fig. 4G.

### Asymmetric friction model: extended angle relations

To explain the emergence of anisotropic friction during both mag-bot extension and bending, we investigated the equilibrated forces acting at the contact edge between each of the mag-bot’s feet and the substrate it resides on, prior to breaking static friction (see Fig. S4 for reference schematics). Force equilibrium in each direction is generally governed by Eq. (3). However the relations for $\alpha{^{\prime}}$ and $\beta{^{\prime}}$ are contingent on the deformation type as documented in Table 3.

**Table 3:** Extended angle relations for model.

Actuation mode	Foot	Deformation type	$\alpha{^{\prime}}$	$\beta{^{\prime}}$
Extension	Hindfoot	II.e	${\theta }_{h}+{\text{tan}}^{-1}\left(\frac{h}{w-u}\right)$	$\frac{\pi }{2}+{\theta }_{h}+{\text{tan}}^{-1}\left(\frac{u}{h}\right)$
Extension	Forefoot	I.e	${\text{tan}}^{-1}\left(\frac{h}{w+u}\right)-{\theta }_{h}$	$\frac{\pi }{2}-{\theta }_{h}-{\text{tan}}^{-1}\left(\frac{u}{h}\right)$
Bending	Hindfoot	I.b	${\theta }_{l}+{\text{tan}}^{-1}\left(\frac{h}{w+u}\right)$	$\frac{\pi }{2}+{\theta }_{l}-{\text{tan}}^{-1}\left(\frac{u}{h}\right)$
Bending	Forefoot	II.b	${\text{tan}}^{-1}\left(\frac{u}{w-u}\right)-{\theta }_{l}$	$\frac{\pi }{2}-{\theta }_{l}+{\text{tan}}^{-1}\left(\frac{u}{h}\right)$

## Supplementary Information

Supplementary Information.

Supplementary Movie S1.

Supplementary Movie S2.

Supplementary Movie S3.

Supplementary Movie S4.

Supplementary Movie S5.

Supplementary Movie S6.

## Data Availability

All data needed to evaluate the conclusions in the paper are present in the paper and/or the Supplementary Materials. Additional data related to this paper may be requested from the authors.
