# Supplementary material for: Locomotion of an untethered, worm-inspired soft robot driven by a shape-memory alloy skeleton
Source: Sci Rep. 2022 Jul 20;12:12392. doi: 10.1038/s41598-022-16087-5 (PMC9300706; doi:10.1038/s41598-022-16087-5)
Supplement: Supplementary file 1 — Supplementary Information. [file 41598_2022_16087_MOESM1_ESM.pdf]

SUPPLEMENTARY MATERIALS

Locomotion of an untethered, worm-inspired soft robot driven by a shape-memory alloy skeleton

Lin Xu[1,2]*, Robert J. Wagner[3]*, Siyuan Liu[1]*, Qingrui He[1], Tao Li[1], Wenlong Pan[1], Yu Feng[1], Huanhuan Feng[1], Qingguang Meng[1], Xiang Zou[1], Yu Fu[1], Xingling Shi[4], Dongliang Zhao[5], Jianning Ding[1†], Franck J. Vernerey[3†]

[1]Institute of Intelligent Flexible Mechatronics, Jiangsu University, Zhenjiang 212013, P. R. China

[2]State Key Laboratory of Solid Lubrication, Lanzhou Institute of Chemical Physics, Chinese Academy of Sciences, Lanzhou 730000, P. R. China

[3]Department of Mechanical Engineering & Material Science and Engineering Program, University of Colorado at Boulder, Boulder 80309-0428, USA

[4]School of Materials Science and Engineering, Jiangsu University of Science and Technology, Zhenjiang 212003, P. R. China

[5]School of Energy and Environment, Southeast University, Nanjing 210096, P. R. China

*These authors contributed equally to this work.

[†]Corresponding author. Email: dingjn@ujs.edu.cn (J.D.); franck.vernerey@colorado.edu (V.F.)

Supplemental Figures Legend:

Figure S1. Design and manufacture of mag-bot.

Figure S2. EDS spectrum of the NiTi SMA.

Figure S3. Magnetothermal deformation response of SMA actuator.

Figure S4. Extended schematics of a wedge undergoing deformation modes I through IV.

Figure S5. Measuring mag-bot speed.

Figure S6. Mechanical characterization of the elastomeric skins.

Figure S7. Movement by a permanent magnet.

Movies Legend:

Movie S1. Movement of mag-bots with different pattern angles. 4 mm-thick mag-bots with skin comprised of EcoFlex 00-30 and pattern angles of 15°, 30°, and 45° are shown walking on level glass substrates for at least three cycles each at 30 × speed.

Movie S2. Mag-bot remains stationary without magnetothermo actuation. A mag-bot residing on a level glass substrate is shown remaining stationary as an induction coil with alternating current running through it passes overhead, thus demonstrating that the mag-bot is not pulled towards the induction coil despite its inclusion of Fe_3O_4 nanoparticles.

Movie S3. Testing the induction coil range. A 4 mm-thick mag-bot with skin comprised of EcoFlex 00-30 and pattern angles of 30° begins centered under a stationary magnetic induction coil. The mag-bot is shown walking at 20 × speed until it is no longer within effective range of the coil, which occurs once the mag-bot has traveled approximately 17 mm.

Movie S4. A 4 mm-thick mag-bot with skin comprised of EcoFlex 00-30 and pattern angles of 15° is shown walking while carrying approximately 1 ×, 2 ×, and 3 × its own body weight.

Movie S5. A 4 mm-thick mag-bot with skin comprised of EcoFlex 00-30 and pattern angles of 30° is shown walking while carrying approximately 1 ×, 2 ×, and 3 × its own body weight.

Movie S6. A 4 mm-thick mag-bot with skin comprised of EcoFlex 00-30 and pattern angles of 45° is shown walking while carrying approximately 1 ×, 2 ×, and 3 × its own body weight.

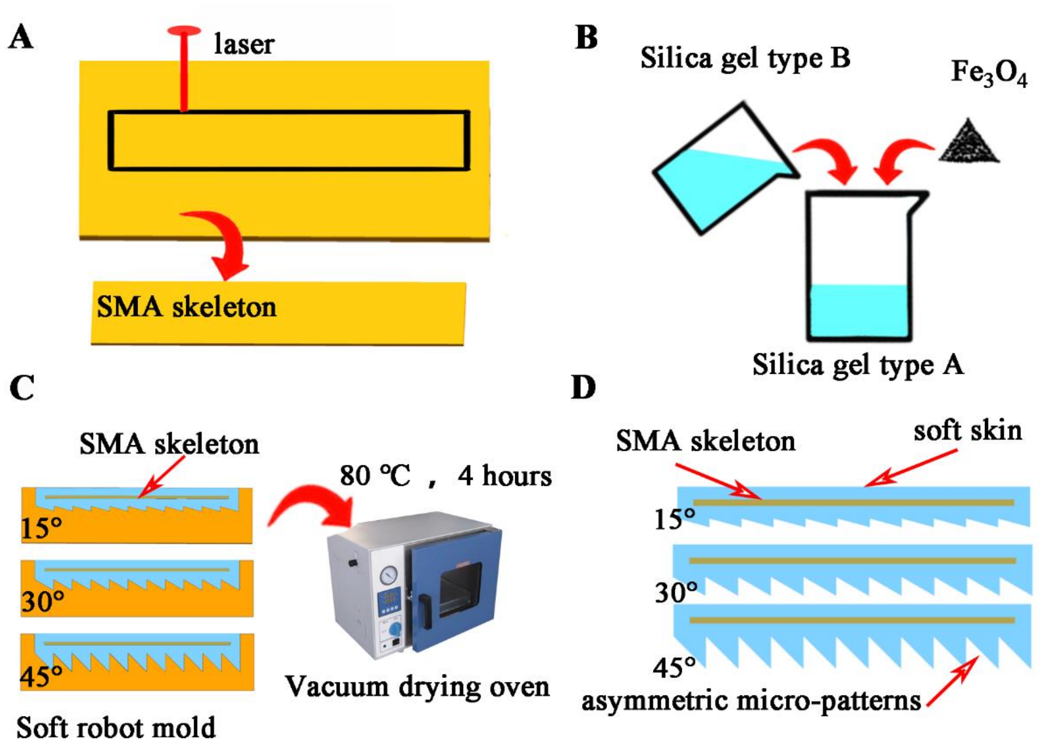

Figure S1. Design and manufacture of mag-bot. (A) Fabrication of SMA skeleton. (B) Fabrication of magnetic silica gel. (C) magnetic skin with asymmetric micro-patterns. (D) Mag-bots with asymmetric micro-patterns of 15°, 30° and 45°.

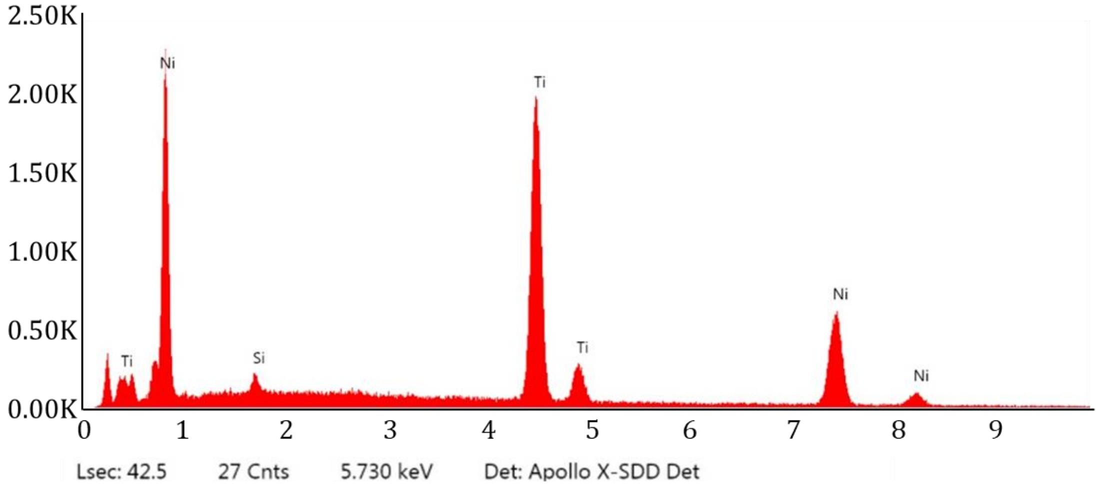

Figure S2. EDS spectrum of the NiTi SMA.

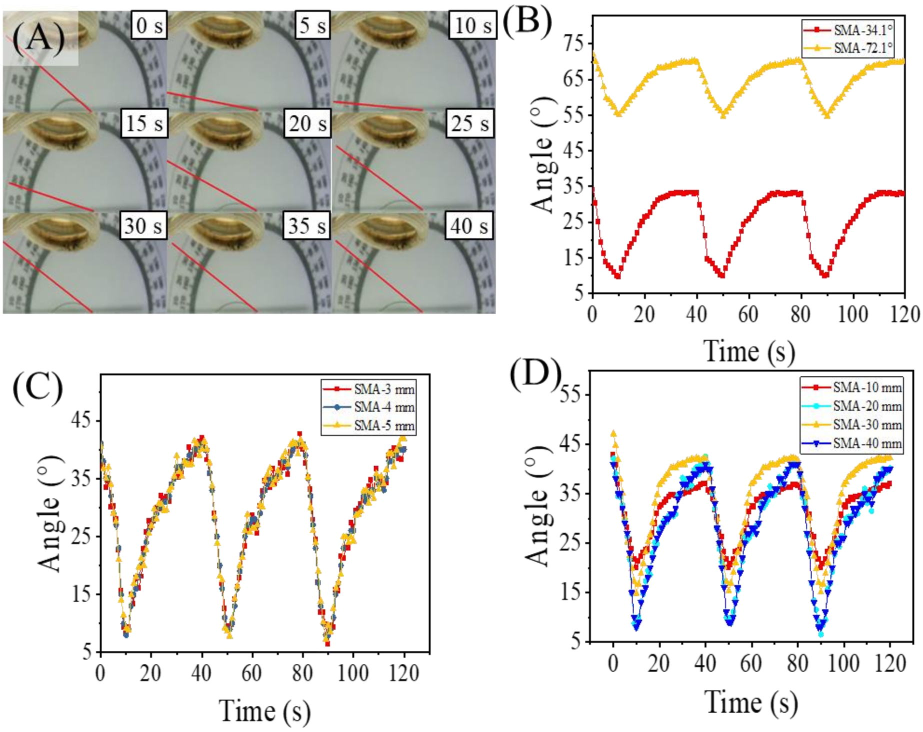

Figure S3. Magnetothermal deformation response of SMA actuator. (A) One cycle of periodic deformation of the SMA (initial bent tangent angle is 34.1°) is displayed, with the tangent angle used to characterize deformation denoted by a red line. **(B)** Deformation angle is plotted with respect to time for SMA skeletons with different initial bent tangent angles undergoing three periods of reversible loading (each). **(C)** Deformation angle is plotted with respect to time for SMA skeletons with different widths undergoing three periods of reversible loading (each). **(D)** Deformation angle is plotted with respect to time for SMA skeletons with different lengths undergoing three periods of reversible loading (each).

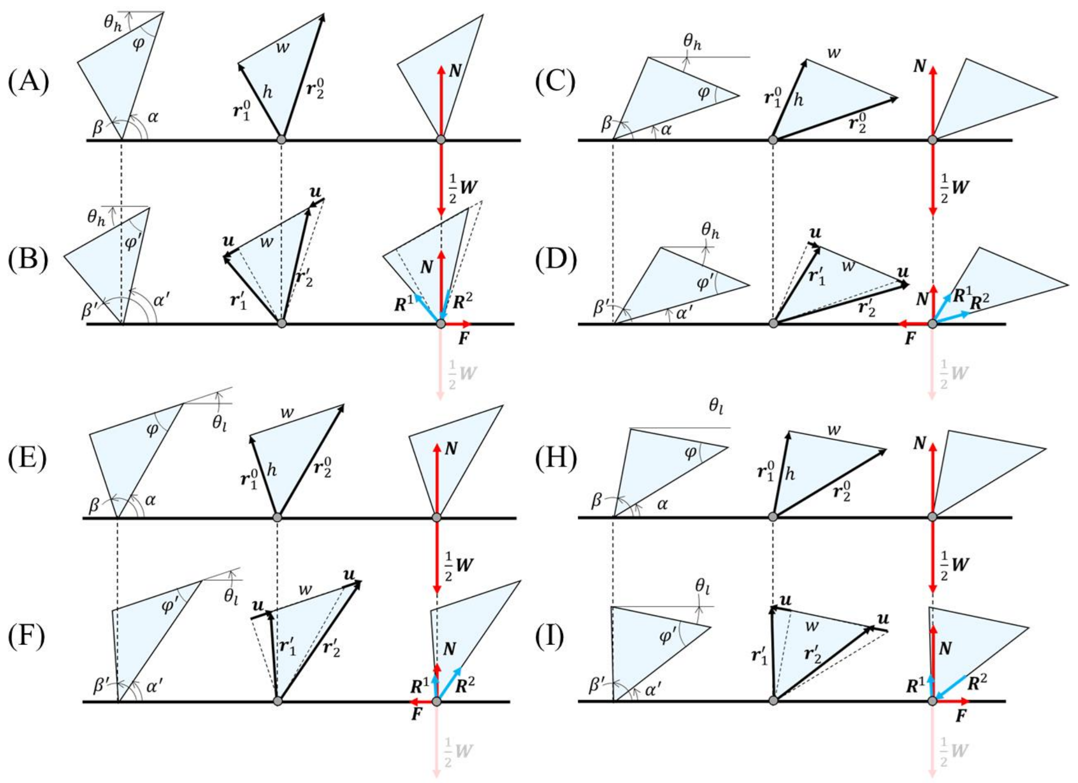

Figure S4. Extended schematics of a wedge undergoing deformation modes I through IV. (A-B) Schematic of Type II.e deformation: a hindfoot wedge during extension. Note that this is reproduced from **Fig. 4C-D** but included here for convenience. **(C-D)** Schematic of Type I.e deformation: a forefoot wedge during extension. **(E-F)** Schematic of Type I.b deformation: a hindfoot wedge during bending. **(H-I)** Schematic of Type II.b deformation: a forefoot wedge during extension. **(A,C,E,H)** depict the wedges just prior to actuation, while **(B,D,F,I)** depict the wedges deformed to the point at which static friction is broken. Leftmost schematics depict the angles referenced in the model, centered schematics depict the spatial vectors, and rightmost schematics depict the forces acting at the contact edge.

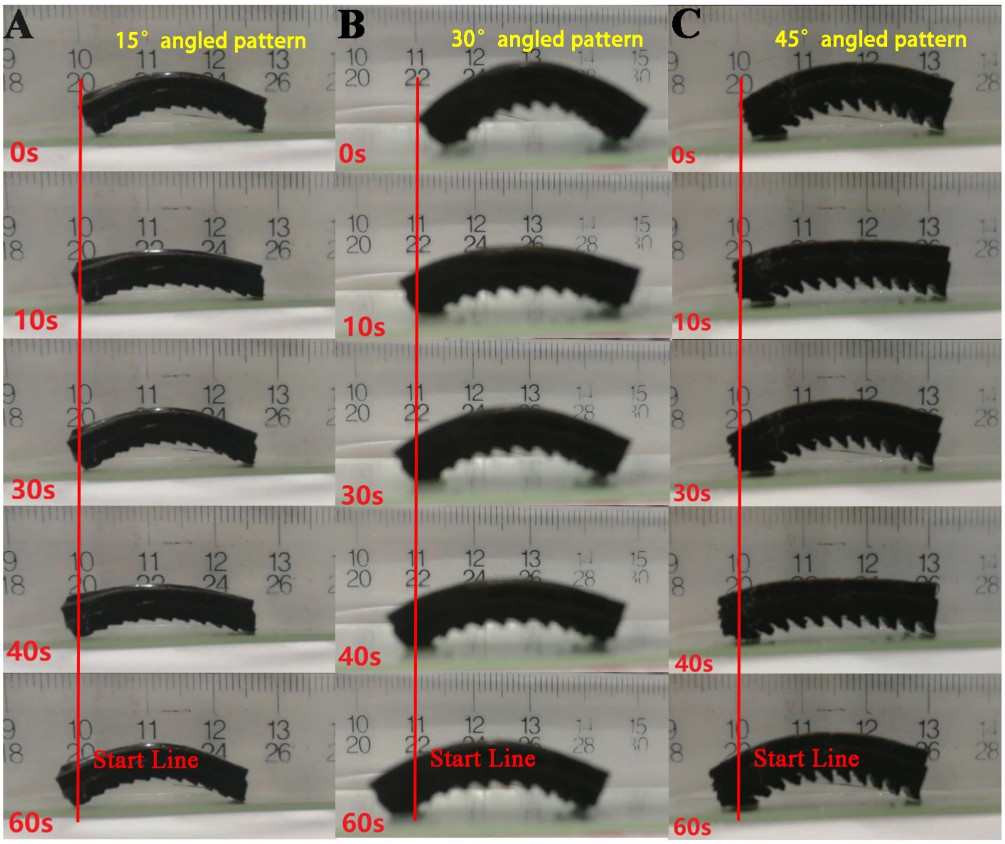

Figure S5. Measuring mag-bot speed. (A) Actual motion state of 15° angled pattern mag-bot. **(B)** Actual motion state of 30° angled pattern mag-bot. **(C)** Actual motion state of 45° angled pattern mag-bot.

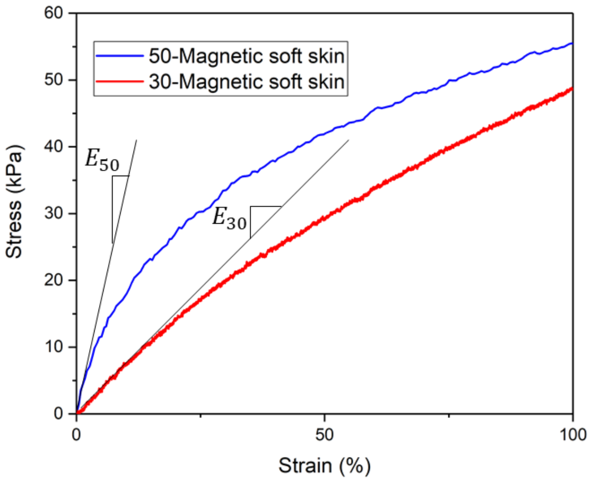

Figure S6. Mechanical characterization of the elastomeric skins. The results of uniaxial tensile tests to 100% strain on samples of EcoFlex 00-30 (red) and EcoFlex 00-50 (blue) are depicted. Although the tangent moduli become comparable above approximately 25% strain due to strain softening of the elastomer (particularly the EcoFlex 00-50), we estimated the modulus at low strains – most closely reflecting the modulus at the start of actuation – as the initial tangent modulus. The moduli of EcoFlex 00-30 and 00-50 at small to intermediate strains ($\sim 0 - 25\%$) were estimated to be on the order of 60-70 kPa and 120-330 kPa, respectively.

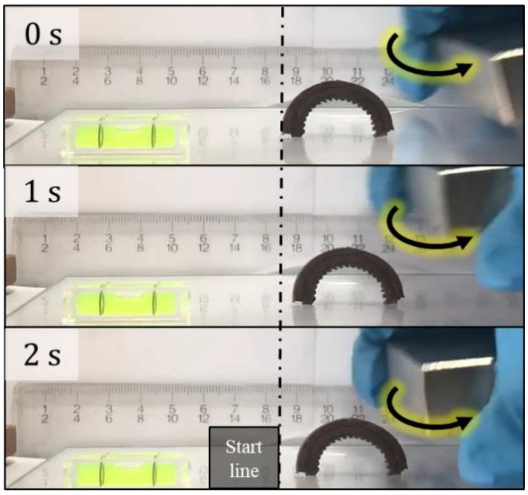

Figure S7. Movement by a permanent magnet. A permanent magnet is repeatedly and closely swept past a mag-bot multiple times over a duration of twos seconds. The highlighted arrow indicates the path of hand movement. Fe_3O_4 particles included in the mag-bot's elastomeric skin cause it to follow the permanent magnet. The magnetism of the elastomeric skin is meant to compliment the magnetothermal actuation of the SMA, in that it may allow users without direct access to the mag-bot to reorient it or help it traverse rough surfaces by using a permanent magnetic field.
